# Health Care Models for Persons with Multiple Chronic Conditions from Populations that Experience Health Disparities: A Scoping Review

**DOI:** 10.1007/s11606-025-09491-w

**Published:** 2025-04-23

**Authors:** Michelle Doose, Simrann Sidhu, Yewande Oladeinde, Dolly Penn White, Lynne S. Padgett, Alicia A. Livinski, Renee Rider, Hwaida Hannoush, Larissa Avilés-Santa

**Affiliations:** 1 Office of Cancer Survivorship, Division of Cancer Control and Population Sciences, National Cancer Institute, Rockville, MD USA; 2 Division of Clinical and Health Services Research, National Institute on Minority Health and Health Disparities, Bethesda, MD USA; 3 National Institutes of Health Library, Office of Research Services, Office of the Director, National Institutes of Health, Bethesda, MD USA; 4 Division of Genomic Medicine, National Human Genome Research Institute, Bethesda, MD USA

**Keywords:** Multiple chronic conditions, Delivery of health care, Health disparities, Patient-centered care, Health services

## Abstract

Almost half of U.S. adults have multiple chronic conditions (MCC), and the prevalence of MCC has significantly increased for racial and/or ethnic minority groups, especially among those aged 45–64 years. Yet, little is known about evidence-based health care models for managing MCC in these populations. The overall objective of this scoping review was to identify the breadth of literature testing health care delivery models or components of models to improve the management of MCC for populations that experience health disparities. The databases of CINAHL Plus, Embase, PubMed, and Scopus were searched for original articles from 2016 to 2023. Included studies had to assess a health care delivery model, intervention, approach, or strategy for improving the management of two or more chronic conditions among U.S. adults. Using Covidence, each record was independently assessed by two reviewers and relevant data about the study, health care model, population studied, and outcomes were extracted. Out of 9583 initially screened records, 17 met the inclusion criteria, of which 5 (29%) were randomized controlled trials. Most (82%) studies focused on the management of psychiatric and physical chronic conditions. The most cited care model was the Patient-Centered Medical Home (41%). Most studies (82%) were conducted within clinical settings: primary care (*n* = 9), specialty care (*n* = 4), and behavioral health (*n* = 2). All studies documented positive improvements in patient outcomes, including fourteen (82%) studies that measured outcomes related to service utilization and eleven (65%) studies that measured clinical outcomes. Four studies (24%) measured cost-related outcomes. While the Chronic Care Model was developed almost 30 years ago, the applicable evidence for MCC is sparse for populations experiencing health disparities. There is an opportunity for research to develop, adapt, integrate, and implement evidence-based health care models for MCC to improve clinically significant health outcomes that align with the patient goal needs.

**Supplementary Information:**

The online version contains supplementary material available at 10.1007/s11606-025-09491-w.

## BACKGROUND

Almost half (42%) of adults in the United States (U.S.) have multiple chronic conditions (MCC),^1^ defined as having two or more chronic conditions such as diabetes mellitus, cardiovascular disease (CVD), chronic obstructive pulmonary disease (COPD), depression, or anxiety. The prevalence of MCC has significantly increased for racial and/or ethnic minorities, especially among those aged 45–64 years.^2,3^ The burden of MCC is twice as high among adults living in poverty,^4^ and there is a significant concentration of MCC in the U.S. continental southeast region.^5^ Despite the accumulation of evidence supporting effective treatments and the development of care guidelines for some of the most common chronic conditions, these advances have not been equitably implemented.^6–9^ The proportion of people achieving treatment and control goals has consistently been lower among populations experiencing health disparities.^10–14^ This often results in high cost of medical services, including emergency department visits and preventable hospitalizations, related to specific chronic conditions^15–27^ and MCC^28–32^ among the uninsured or underinsured^33^ and those in rural communities.^33,34^

The management of MCC presents unique challenges beyond a single chronic condition, such as treatment conflicts, complex care coordination, care prioritization, and self-management burden.^35^ Evidence-based health care models implemented at the health system or clinic level could be leveraged to address these challenges that unduly impact populations and communities. Health care models refer to the systematic organization and delivery of high-quality patient-centered care.^36,37^ For example, the Chronic Care Model emphasizes the assurance of productive interactions between the patient and care team and proposes the integration of six elements: health care organization, community resources, patient self-management support, delivery system design, health care provider decision support, and clinical information system.^38^ Other health care models in which components of the Chronic Care Model have been expanded or enhanced include the Patient-Centered Medical Home,^39,40^ eHealth Enhanced Chronic Health Care Model,^41^ Community-Based Transition Model,^42^ Model for Developing Complex Interventions in Nursing,^43^ Home-Based Model,^44^ Integrated Delivery Systems Model,^45^ Team-Based Care,^46^ Family Management Framework,^47^ and others.^48–50^ Recently, the Value-Based Care Model has gained increasing interest.^51–53^ Furthermore, evidence-based multiple disease-specific guidelines that account for the concurrent treatment of MCC are needed as they presently do not exist.^54^ While some health care models have demonstrated improvements in health outcomes in a single condition, we are unaware if these models have been shown to improve optimal management and control of two or more coexisting chronic conditions specifically for populations that experience health disparities.

Therefore, we performed a scoping review of the literature over an eight-year period to identify studies of health care models for persons with MCC from populations that experience health disparities and to determine which health care models or components of these models reduced health disparities. We were particularly interested in studies that tested and described health care models that successfully implemented evidence-based care or practice guidelines for at least two coexisting chronic conditions, especially within the context of low-resource settings or settings serving a high need population.

## METHODS

We chose the scoping review approach to comprehensively map the current literature and identify research gaps in this field.^55^ We used the Preferred Reporting Items for Systematic reviews and Meta-Analyses extension for Scoping Reviews (PRISMA-ScR) Checklist to report this review (Appendix 1).^56^

### Eligibility Criteria

Eligible studies had to be conducted in the U.S. with adults aged 18 years and older. At least one of the conditions in the study had to be a physical health condition and both chronic conditions had to be independent and not a consequence of each other (e.g., diabetes mellitus and diabetic neuropathy). Analytical studies, with or without a comparison group, were included if they assessed any health care model, intervention, approach, or strategy for improving the management or coordination of comprehensive health care and outcomes of at least two coexisting chronic conditions. Outcomes had to be related to the MCC, including any process of care (e.g., hemoglobin A1 C (A1 C) recommendation or Patient Health Questionnaire- 9 (PHQ- 9) testing frequency) or health outcome measured at the patient level (e.g., attaining A1 C recommended level or PHQ- 9 score). Examples of eligible chronic conditions, along with the detailed screening criteria, are stated in Appendix 2.

During the full-text review screening, studies were included if at least half of the study population was from a population experiencing health disparities or if the study was conducted in a health care setting that served such a population (e.g., rural community health center or Medicaid serving clinic). We used the National Institutes of Health (NIH) designated populations experiencing health disparities as of 2023,^57^ which includes racial and/or ethnic minority groups (i.e., American Indian or Alaska Native, Asian, Black or African American, Latino or Hispanic, Native Hawaiian and Pacific Islander), socioeconomically disadvantaged populations (e.g., Medicaid beneficiaries), sexual and gender minority groups, rural underserved communities, and persons with disabilities.

### Information Sources and Search

A biomedical librarian (AAL) conducted literature searches using four electronic databases: CINAHL Plus (Ebscohost), Embase (Elsevier), PubMed (US National Library of Medicine), and Scopus (Elsevier). A combination of keywords and controlled vocabulary (e.g., CINAHL Subject Headings, EMTREE, and MeSH) for each concept (e.g., chronic condition, health care model, United States) was created by the biomedical librarian with input from the review team members. An initial search was conducted in March 2022 with an update done in January 2024, and a revised search with additional search terms completed in February 2024. The original search was planned as a six-year review to capture the most current literature but was extended two additional years to add search terms to further explore health care models (e.g., integrated care, patient group care, mobile clinics) identified from the screening of the first set of results; the other parts of the search strategy for the other concepts remained the same. The final search strategies for the original and revised searches are in Appendix 3. The searches were limited to those published from January 2016 through December 2023 in English and excluded publication types specified in the exclusion criteria. All database results were exported to EndNote 20 (Clarivate Analytics), duplicates identified, and unique records exported to Covidence (Veritas Health Innovations) for screening.

### Selection of Sources of Evidence

Two-levels of screening were completed in Covidence. First the titles and abstracts were screened independently by two reviewers using the eligibility criteria. Second, records that proceeded to full-text review were screened independently by two reviewers. Discrepancies between reviewers were arbitrated by a different third reviewer for both steps of screening. The title and abstract screening procedures were tested on a random set of records to ensure a shared mental model and interpretation of eligibility and exclusion criteria among reviewers.

### Data Collection and Data Items

Covidence was used for data collection. Two reviewers independently collected the data from each included article. A third reviewer made the final decision on any data discrepancies identified using Covidence’s consensus feature. Before commencing data collection, the data collection form, data items, and process was tested by the reviewers and then further refined.

The following data items were collected: year published, title, study aims, study design, population description, inclusion and exclusion criteria, total number of participants, populations of interest using NIH definitions, domain of MCC (i.e., psychiatric and physical conditions or only physical conditions), list of chronic diseases studied, name of health care model, type of model (i.e., chronic care model, patient-centered medical home, collaborative care, integrated care, or other), elements of health care model or component(s) being addressed,^58^ length of follow-up, description of health care setting, comparator group, selection and description of outcomes measured (i.e., clinical, service utilization, function status, quality of life, feasibility, sustainability, other), level of outcomes measured, article explicitly state clinically significant improvement, clinical threshold, and report proportion of population that achieved optimal clinical outcomes, and summary of key findings.

### Statistical Analysis

The data collected in Covidence was downloaded and imported into SAS, version 9.4 (SAS Institute, Cary, NC) to calculate and report descriptive statistics of the included studies. A summary table of the included studies listed by study design (i.e., randomized controlled trials (RCTs) versus non-randomized) was created and frequencies and percentages were calculated for the key variables extracted.

## RESULTS

### Study Selection

**Figure 1:**
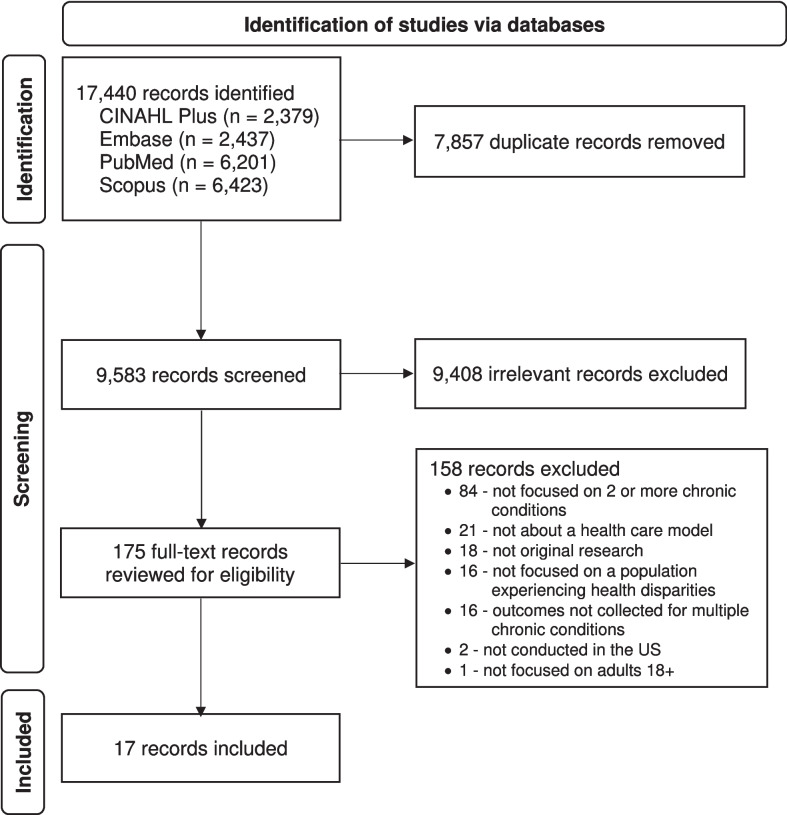
PRISMA flow diagram of included and excluded studies.

The searches identified 17,440 records, which are outlined in the PRISMA flow diagram in Fig. 1. After deduplication, the titles and abstracts of 9583 records were screened. Of these, 9408 were excluded and 175 records screened at the full-text level. Most exclusions were due to not being focused on the management of two or more coexisting chronic conditions (*n* = 84) followed by not being about a health care model (*n* = 21). After full-text review, 17 records were included in the final analytic data set (Tables 1 and 2).

**Table 1:** Included Studies by Study Design (*n* = 17)

First author last name, year published	Health care model	Model components and health care setting	Chronic conditions studied	Number enrolled	Length of follow-up	Patient outcomes
Randomized controlled trial (*n* = 5, 29%)						
Chan, 2023^59^	Streamlined Unified Meaningfully Managed Interdisciplinary Team (SUMMIT)	Co-located medical and behavioral multidisciplinary primary care team at a FQHC	Two or more chronic medical conditions (e.g., diabetes, CHF, or liver disease) or chronic condition and SUD or mental illness	159	6 months	= Hospitalization = ED use + Primary care visits + Behavioral health visits + Feasibility = Patient-reported outcomes (activation experience, HRQOL) + Self-rated health
Shah, 2023^60^	Diabetes Research, Education, and Action for Minorities (DREAM) Atlanta intervention	Telehealth intervention with health education sessions and action plan development with community health workers	Diabetes and hypertension	183	6 months	+ Hypertension control = HbA1c level + Weight loss + Feasibility (retention rate)
Felker, 2022^61^	Technologies to improve drug Adherence and Reinforce Guideline-based Exercise Targets in patients with Heart Failure and Diabetes Mellitus (TARGET-HF-DM) trial	Telehealth intervention with pillbox and text messages for medication management teaching at 6 clinical sites	Heart failure and diabetes	187	6 months	+ Physical activity + Quality of life* = Medication adherence = Heart failure clinic status = Glycemic control (HbA1c)
Junkins, 2021^62^	Telemedicine-administered, culturally adapted cognitive behavioral therapy for depression and antiretroviral therapy adherence (CBT-AD) approach	Telemedicine-delivered cognitive behavioral therapy at 4 HIV care outpatient clinics	HIV and depression	22	6 months	= Feasibility/acceptability = Depression symptoms = Viral load suppression = Treatment adherence
Errichetti, 2020^63^	Reverse Co-located Integrated Care	Multidisciplinary care team for medical care at a behavioral health clinic	Serious mental illness (bipolar, schizophrenia, depression, or a combination of these diagnoses) and comorbid chronic disease (diabetes)	416	12 months	+ Hypertension control + Glycemic control (HbA1c) + Body mass index = Total cholesterol = Depressive symptoms (PHQ- 9 score)
Non-randomized clinical trial (*n* = 12; 71%)						
Steinman, 2023^64^	Program to Encourage Active, Rewarding Lives (PEARLS)	Home-based care program developed with social service organization for self-management, psychoeducation, support, coordination, and mental health specialty care	Depression and another comorbidity: CVD, diabetes, gastrointestinal, nervous system, pulmonary, renal, SUD	164	24 months	+ Inpatient hospitalizations = ED visits + Nursing home days + 12-month all-cause mortality = Depression score = Self-rated health
Germack, 2022^65^	Patient-Aligned Care Team (PACT) initiative	Team-based care implementation within the Veterans Health Administration (831 primary care outpatient clinics)	Mental health condition (MDD, psychosis/schizophrenia, bipolar disorder, anxiety, PTSD, SUD) and a comorbid physical health condition (chronic pain or arthritis, hypertension, COPD, diabetes, thyroid disorders, CAD, cardiac arrhythmias)	1,444,942	12 months	+ Hospitalization rates
Grove, 2020^66^	Community Care of North Carolina (CCNC) program	Medical home enrollment within primary care practices to improve primary care access	Schizophrenia and/or depression, and diagnosis of hypertension, hyperlipidemia, seizure disorder, COPD, diabetes, or asthma	83,819	36 months	+ ED use + Psychiatric facility inpatient stays + Outpatient visits + Medical expenditures
Schuttner, 2020^67^	Patient-Aligned Care Team (PACT) initiative	Team-based care, care management, and expanded access and care within the Veterans Health Administration 900 + primary care clinics	≥ 3 chronic diseases in ≥ 3 body systems (hypertension, diabetes, depression, ischemic heart disease, alcohol use disorder, CHF)	318,764	12 months	+ Quality metric for those with 3–4 chronic conditions + Glycemic control + Lipid control = Depression screening
Irwin, 2019^68^	Bridge Intervention: Person-Centered Collaborative Care for Serious Mental Illness and Cancer	Team care with psychiatry and cancer care at a cancer center	SMI (schizophrenia, schizoaffective disorder, bipolar disorder, or MDD with prior psychiatric hospitalization) and cancer (thoracic, gastrointestinal, breast, or head and neck)	25	3 months	+ Psychiatric illness severity (BPRS) = Depression symptoms (PHQ- 9) = Quality of life + Feasibility/acceptability + Implementation
Crits-Christoph, 2018^69^	Pennsylvania Chronic Care Initiative (CCI)	Practice transformation with coordination processes for behavioral and medical care at 137 primary care practices	HIV and one of 4 comorbid chronic medical conditions (diabetes, COPD, asthma, and CHF) and comorbid behavioral health conditions psychiatric (MDD, schizophrenia/ schizoaffective disorder, bipolar disorder, PTSD, and anxiety disorders) and/or SUD	2879	36 months	= ED use + All inpatient services = All outpatient services + Total inpatient + Total cost savings
Gilmer, 2018^70^	Behavioral Health Integration and Complex Care Initiative (BHICCI)	Practice transformation including collaborative and complex care management within health systems and organizations including FQHC (primary care), multispecialty clinics, and behavioral health clinics	Severe mental illness – schizophrenia, bipolar disorder, and severe major depression; diabetes, obesity, hypertension	6699	12 months	+ Systolic blood pressure + HbA1c level + Depression (PHQ- 9) + Body mass index + Cost savings (inpatient only)
Matzke, 2018^71^	Pharmacist–physician collaborative care model	Team-based pharmacist– physician collaborative care approach implemented at 6 hospitals and 22 PCMH practices within a single health system	CHF, hypertension, hyperlipidemia, diabetes mellitus, asthma, COPD, and depression	4960	12 months	+ HbA1c + Blood pressure = LDL = Total Cholesterol = ED use + Hospitalizations + Cost savings
Swietek, 2018^72^	Community Care of North Carolina (CCNC) Patient-Centered Medical Home Program	PCMH program that provides primary care, specialty care coordination, and care management within 14 not-for-profit care networks with 1600 primary care practices	Asthma, COPD, diabetes, hypertension, hyperlipidemia, seizure disorder, MDD, and schizophrenia	131,036	12 months	+ Diabetes metrics: HbA1c testing, attention for nephropathy, liver function, eye exam, lipid panel, medication + Lipid testing + Psychotherapy – short-acting b-agonists for asthma
Taber, 2018^73^	Pharmacist-led, Technology-Aided, Education Intervention	Pharmacist-led encounters at clinic with telehealth monitoring of glucose and blood pressure home devices	Kidney transplant recipients with diabetes and hypertension	60	6 months	= Blood pressure control* = HgA1c < 7%* = LDL, triglycerides, or HDL + Medication errors reduction = Medication adherence
Rhodes, 2016^74^	Pennsylvania Chronic Care Initiative (CCI)	PCMH enrollment at 96 primary care practices	Chronic medical condition—diabetes, COPD, asthma, heart failure; Comorbid behavioral health conditions—psychiatric (MDD, schizophrenia/schizoaffective disorder, bipolar disorder, PTSD anxiety disorders) and SUD (opioid, cocaine, alcohol)	22,210	12 months	+ ED visits + Inpatient psychiatric utilization + Cost savings
Rossom, 2017^75^	Care of Mental, Physical and Substance use Syndromes (COMPASS) initiative	Intensive care management using treat-to-target guidelines of care at 172 primary care clinics within 18 health systems (integrated health systems, FQHCs, multisite physician practices, and individual practice associations)	Depression and diabetes or CVD	3363	11 months	+ Depression severity (PHQ9 score < 5) + HbA1c goal < 8.0% + Feasibility (depression care satisfaction) = Hypertension control (< 140/< 90 mmHg) = Feasibility (satisfaction)

*Abbreviations*: *BPRS*, Brief Psychiatric Rating Scale; *CAD*, coronary artery disease, *CHF*, chronic/congestive heart failure; *COPD*, chronic obstructive pulmonary disorder; *CVD*, cardiovascular disease; *ED*, emergency department; *FQHC*, Federally qualified health center; *HIV*, human immunodeficiency virus; *HDL*, high-density lipoprotein; *HRQOL*, health-related quality of life; *LDL*, low-density-lipoprotein; *PCMH*, patient-centered medical home; *PTSD*, posttraumatic stress disorder; *MDD*, major depressive disorder; *SUD*, substance use disorder

+ improvements in outcomes, = no changes in outcomes, – worse outcomes, * study assessed clinical significance

**Table 2:** Characteristics of Included Studies (*n* = 17)

	*n*	%
Types of health care model*		
Patient-centered medical home	7	41.18
Collaborative care model	5	29.41
Chronic care model	3	17.65
Integrated care model	2	11.76
Other	5	29.41
Elements of the health care model*		
Health system	10	58.82
Community resources	3	17.65
Delivery system design	14	82.35
Self-management support	7	41.18
Decision support	4	23.53
Clinical information systems	5	29.41
Study populations*		
Lower socioeconomic status	13	76.47
Racial and ethnic minority groups	9	52.94
Rural underserved or rural clinic	6	35.29
People with disabilities	2	11.76
Types of comorbid conditions studied*		
Diabetes	15	88.24
Cardiovascular disease	8	47.06
Hypertension	8	47.06
Serious mental illness	8	47.06
Depression	7	41.18
Heart failure	7	41.18
Chronic obstructive pulmonary disease	6	35.29
Asthma	5	29.41
Substance use disorder	5	29.41
Hyperlipidemia	3	17.65
Liver disease	2	11.76
Human immunodeficiency virus	2	11.76
Renal disease	2	11.76
Cancer	1	5.88
Outcomes measured*		
Service utilization	14	82.35
Clinical outcomes	11	64.71
Quality of life	4	23.53
Feasibility	5	29.41
Cost	4	23.53
Sustainability	1	5.88
Level outcomes measured*		
Patient	17	100.00
Health system(s)/clinic(s)	6	35.29
Care team	2	11.76
Societal/policy	1	5.88
Clinically significant improvements stated		
Yes	3	17.65
No	14	82.35

*More than one option could be selected. Therefore, percent column will not sum to 100%

### Characteristics of Included Studies and Multiple Chronic Conditions

Among the 17 included studies, 71% (*n* = 12) were observational study designs (e.g., non-randomized quasi-experimental or cohort studies) and 29% (*n* = 5) were RCTs (Table 1). Most (82%) studies focused on the management of one psychiatric and one physical chronic condition while three studies focused exclusively on two physical chronic conditions, specifically diabetes mellitus and CVD. The most common psychiatric condition of study was serious mental illness (*n* = 8), including major depressive disorder, schizophrenia, bipolar disorder, and posttraumatic stress disorder (Table 2). Five studies also included substance use disorder. Diabetes mellitus (*n* = 15), CVD (*n* = 8), hypertension (*n* = 8), and COPD (*n* = 6) were the most common physical chronic conditions studied.

### Characteristics of the Health Care Model and Health Care Setting

The Patient-Centered Medical Home was the most cited health care model (*n* = 7) followed by the Collaborative Care Model (*n* = 5) and the Chronic Care Model (*n* = 3) (Tables 1 and 2). Four other health care models used telehealth (e.g., mHealth, technology). Most studies (82%) focused on the delivery system design through team-based care or case management followed by addressing the health system (59%) by co-locating services such as primary care and behavioral health. The integration of community resources (18%) was the element least addressed. Health care settings were diverse and included hospitals, primary care networks, federally qualified health centers, multispecialty clinics, and behavioral health clinics as well as telehealth. Three studies were conducted within health systems and 14 studies were conducted within the clinical setting, including primary care (*n* = 9), specialty care (*n* = 4), and behavioral health (*n* = 2). Two studies occurred within the Veterans Administration health care system.

### Characteristics of the Study Population

Participants enrolled in these studies ranged from 22 to 1,444,942 persons with a mean follow-up of 12 months. Thirteen studies (76%) included populations of lower socioeconomic status, including Medicaid beneficiaries (*n* = 8) and people experiencing homelessness (*n* = 2). Nine studies (53%) included or focused on racial and/or ethnic minority groups. Six studies included rural populations or clinics within rural communities. Two studies (12%) included people with disabilities.

### Study Outcomes

All studies demonstrated improvements in outcomes associated with the health care model assessed (Table 1). Fourteen (82%) studies measured outcomes related to service utilization, such as rates of hospitalizations, emergency room visits, primary care visits, as well as receipt of testing for specific conditions. Clinical outcomes (65%) were also commonly measured, including blood pressure level, A1 C level, depressive symptoms, and lipid testing. Of note, most studies on diabetes mellitus focused on glycemic control but not on other clinical goals and recommended guidelines of care. Four studies (24%) measured outcomes related to cost savings and cost expenditures. Five studies assessed feasibility and only one assessed sustainability. All studies measured patient-level outcomes, and six studies measured outcomes at the clinic or health system level.

## DISCUSSION

The aim of this scoping review was to identify the breadth of literature testing health care models or components of models to improve the management of MCC for populations that experience health disparities. While there have been other published reviews on MCC,^76–80^ to our knowledge this is the first review to evaluate health care models specifically for the management of two or more coexisting chronic conditions for these populations. We identified 17 studies over an eight-year period from the literature. Research on the management of one physical and one psychiatric health conditions was more common than for two physical health conditions. The Patient-Centered Medical Home was the most cited health care model, with most studies focused on the delivery of team-based care or case management followed by addressing the organization of care delivered at the health system level. Most interventions occurred within a clinic setting, with a particular focus in primary care. Populations of focus included persons from lower socioeconomic status and racial and/or ethnic minority groups. While health care models for individual chronic diseases were developed almost 30 years ago,^81^ the applicable evidence for MCC remains sparse.

This was not a systematic review, and therefore the effectiveness of the models and study quality were not assessed. From the extensive literature search, we identified five unique health care models tested for persons with MCC from populations that experience health disparities. The integrated care and telehealth models were the only models tested in RCTs. Although positive patient outcomes were reported among the 17 studies, the study characteristics and outcomes measured across studies varied. For example, only three studies assessed clinical significance of changes in patient-level outcomes,^61,70,73^ of which two studies compared outcomes by race and ethnicity.^73,75^ Specifically, Taber et al. telehealth intervention reported an 18% increase of the study population reaching a blood pressure target < 140/90 mmHg, which was more significant among African American participants than in non-African American participants.^73^ On the other hand, there were no significant changes in the percent attaining blood pressure < 130/80 mmHg. However, African American participants did experience statistically significant reductions in both systolic and diastolic blood pressure compared to non-African American participants. They also reported a 14% increase among those reaching A1 C < 7%, which was significant among non-African American participants only. Both African American and non-African American participants experienced reductions in A1 C similar in magnitude. Gilmer et al. collaborative care study reported statistically significant improvements in systolic blood pressure, A1 C, body mass index, and the PHQ- 9 score at follow-up among those who exceeded the initial screening threshold.^70^ Those improvements did not reach clinical significance among any of the racial and/or ethnic groups represented in the study. Using the Kansas City Cardiomyopathy Questionnaire to assess quality of life, Felker et al. telehealth RCT demonstrated clinically significant improvement in 51% of patients receiving a mobile health intervention designed to increase guideline-based goals, which was 12% higher compared to the control group.^61^

Additionally, we would expect health care models for MCC to be tailored for specific populations given the undue burden of social determinants of health. Three studies centered on specific racial and/or ethnic minority groups, including South Asian immigrants,^60^ African American women,^62^ and Hispanic population.^63^ Two additional studies addressed social needs along with the management of MCC. Steinman et al. was the only home-based collaborative care model developed with trusted social service organizations to address non-clinical care issues such as housing and food insecurity.^64^ Chan et al. specifically addressed the needs of individuals with MCC experiencing homelessness by implementing a co-located multidisciplinary team that included care management for social needs.^59^ Strengthening the social and clinical care integration for patients with MCC who are also experiencing transportation, financial hardship, inadequate housing, food insecurity, or cultural/language barriers is a critical area for future research.^82^

The limited or inconsistent efficacy or effectiveness of different health care models on the improvement of clinical outcomes in persons with MCC has been documented in other reviews and in the global literature. For instance, Barajas-Nava et al. systematic review of 25 RCTs in health care models for adults aged 60 + with MCC reported that most of these interventions were focused on education, and mostly in the domain of patient-clinician communication, and that 57% were ineffective at providing any benefits.^79^ Eriksen et al. assessed the effectiveness of 20 RCTs on health-related quality of life, mental health, and mortality.^80^ The barriers to fully implementing or integrating various health care models globally have also been documented. Longhini et al. highlighted the need to assess caregivers-related outcomes and clearer description of interventions in organizational models of pediatric primary care worldwide.^83^ Ludwick et al. underlined the readiness required, especially financial and resources, which is key for a successful implementation of community health worker-led integrated care in Ethiopia.^84^ Pinter et al. described financial barriers, shortage of professionals, and lack of training as the main barriers to the implementation of integrated models of care in Asia, whereas financial incentives seemed to increase their success.^85^ The authors also observed that performance assessments were rarely embedded in the models. Wilson et al. highlighted the very limited studies of health care models for persons with MCC in Canada, and the call for patient-centered guidelines of care, transdisciplinary care teams, and research.^86^ Rohwer et al. pointed out the worldwide heterogeneity of integrated models, with inconsistent integration and measured outcomes.^87^ These examples denote universal challenges revolving around the increasing prevalence and awareness of MCC, and invoke comprehensive rethinking of approaches to prevent, diagnose, and effectively treat MCC that both consider the cost-effective or cost-saving approaches for long-term sustainability.

Considering the complexities of health care delivery and population health, we highlight important future directions. Research is needed to better understand how to organize and deliver care for MCC to attain optimal health outcomes based on evidence-based care guidelines and patient-centered goals. However, the exclusion of persons with MCC from clinical trials precludes the generation of necessary evidence that would inform the optimal integration of new treatments into real-life clinical settings.^37,88,89^ Health care models should be adapted to patients’ health care needs, personal goals, resources, and limitations. Examples of patient-centered goals or personal health outcomes may include what the patients hope to achieve, affordability and out-of-pocket expenses, lost wages, time burden for health care, physical and mental function and independence, well-being, life expectancy, social and occupational engagement, burden of multiple diagnostic and surveillance tests, procedures, or treatment, and the complexity of self-management tasks.^90^ The lack of focus on reducing health disparities highlights an opportunity area for future development and tailoring of health care models. Examples include the creation of high-functioning interprofessional care teams that can effectively coordinate care within and between care settings and during care transitions,^91^ the integration of supportive personnel in a comprehensive and holistic care plan,^92^ and the development of algorithms, decision support tools, and telemedicine technology for use by clinicians, patients, and caregivers to engage in shared decision-making and communication.^93^ Furthermore, understanding how best to implement these models across complex and diverse health care settings, including assessing its feasibility, affordability, and sustainability, would be critical for their long-term success and to have implications for policy and planning.^94^ As noted, only three studies reported on clinical significance, and those studies did not achieve optimal outcomes for all patients in their study population. Methods and measures of determining delivery of optimal health care and achieving health outcomes for all are also needed when health care models are tested while also balancing the individual care needs for different patients.^95,96^

Several limitations should be noted when interpreting our findings. First, despite a robust search strategy of peer-reviewed published articles that was inclusive of many chronic conditions, it is possible that some relevant articles were not identified or missed. While many articles were screened in this review, many failed to manage two or more chronic conditions, often focused on managing one condition among populations with multiple conditions. This strict inclusion criteria limited the scope of our findings, but also highlights the dearth of research. Additionally, ten studies included many chronic conditions in their inclusion criteria rather than focusing on the management of two or three conditions, which made the interpretation of findings for specific co-occurring conditions difficult. Lastly, at least half of the study population had to be from a NIH-designated population that experiences health disparities or be in a health care setting that served such population. However, not all articles clearly described their study population and as such, we only included articles if the population sociodemographic characteristics explicitly aligned with our search criteria. Despite these limitations, the literature on health care models for MCC is emerging,^76^ and our scoping review is one of the first to highlight the opportunities to advance the science and can be used to inform future reviews with a formal quality evaluation.

While there has been a call for a fundamental change in the care for persons with MCC over the last several decades, health care delivery research is nascent for the management of multiple, coexisting chronic conditions, especially among populations that experience health disparities. Our ability to advance health care delivery research and population health is constrained when research studies do not reflect the real-world experiences of populations that experience health disparities and MCC. There is an opportunity for multilevel, multicomponent, innovative research to develop adapt, integrate, and implement evidence-based health care models or strategies for MCC to improve clinically significant health outcomes that align with patient goal needs.

## Supplementary Information

Below is the link to the electronic supplementary material.

Supplementary file1 (PDF 584 KB)

Supplementary file2 (DOCX 31 KB)

Supplementary file3 (DOCX 92 KB)

## References

[1] **Buttorff C, Ruder T, Bauman M.** Multiple chronic conditions in the United States. Available at: https://www.rand.org/pubs/tools/TL221.html. Accessed 3 July 2024.

[2] **Freid VM, Bernstein AB, Bush MA.** Multiple chronic conditions among adults aged 45 and over: trends over the past 10 years. NCHS Data Brief. 2012 (100):1-8. 23101759

[3] **Zhang Y, Misra R, Sambamoorthi U.** Prevalence of multimorbidity among Asian Indian, Chinese, and Non-Hispanic White adults in the United States. Int J Environ Res Public Health. 2020;17(9):3336. 32403412 10.3390/ijerph17093336 PMC7246600

[4] **Boersma P, Black LI, Ward BW.** Prevalence of multiple chronic conditions among US Adults, 2018. Prev Chronic Dis. 2020;17:E106-E. 32945769 10.5888/pcd17.200130 PMC7553211

[5] **Benavidez G, Zahnd W, Hung P, Eberth J.** Chronic disease prevalence in the US: sociodemographic and geographic variations by zip code tabulation area. Prev Chronic Dis. 2024;21:E14. 10.5888/pcd21.230267 PMC10944638 38426538

[6] American Diabetes Association Professional Practice Committee. Standards of care in diabetes-2024. Diabetes Care. 2024;47(Suppl 1):S1-s308. 10.2337/dc24-S017 PMC10725796 38078588

[7] American College of Cardiology. Guidelines & clinical documents. Available at: https://www.acc.org/Guidelines. Accessed 3 July 2024.

[8] **Arnold MJ.** Treatment of chronic obstructive pulmonary disease: Guidelines from the American Thoracic Society. Am Fam Physician. 2021;104(1):102-3. 34264596

[9] American Psychiatric Association. Clinical practice guidelines. Available at: https://www.psychiatry.org/psychiatrists/practice/clinical-practice-guidelines. Accessed 24 July 2024.

[10] **Aggarwal R, Chiu N, Wadhera RK, et al.** Racial/ethnic disparities in hypertension prevalence, awareness, treatment, and control in the United States, 2013 to 2018. Hypertension. 2021;78(6):1719-26. 34365809 10.1161/HYPERTENSIONAHA.121.17570 PMC10861176

[11] **Zakaria NI, Tehranifar P, Laferrère B, Albrecht SS.** Racial and ethnic disparities in glycemic control among insured US adults. JAMA Netw Open. 2023;6(10):e2336307. 37796503 10.1001/jamanetworkopen.2023.36307 PMC10556965

[12] **Akinbami LJ, Liu X.** Chronic obstructive pulmonary disease among adults aged 18 and over in the United States, 1998–2009. In: National Center for Health Statistics, editor. no 63 ed. Hyattsville, MD NCHS data brief; 2011. 22142836

[13] **McGregor B, Li C, Baltrus P, et al.** Racial and ethnic disparities in treatment and treatment type for depression in a national sample of Medicaid recipients. Psychiatr Serv. 2020;71(7):663-9. 32237981 10.1176/appi.ps.201900407 PMC8842821

[14] **Zavala VA, Bracci PM, Carethers JM, et al.** Cancer health disparities in racial/ethnic minorities in the United States. Br J Cancer. 2021;124(2):315-32. 32901135 10.1038/s41416-020-01038-6 PMC7852513

[15] **Aratani Y, Nguyen HA, Sharma V.** Asthma-related emergency department visits among low-income families with young children by race/ethnicity and primary language. Pediatr Emerg Care. 2020;36(11):e636-e40. 30672895 10.1097/PEC.0000000000001430

[16] **Arcoleo KJ, McGovern C, Kaur K, et al.** Longitudinal patterns of Mexican and Puerto Rican children’s asthma controller medication adherence and acute healthcare use. Ann Am Thorac Soc. 2019;16(6):715-23. 30860858 10.1513/AnnalsATS.201807-462OC PMC6543480

[17] **Casten R, Rovner B, Chang AM, et al.** A randomized clinical trial of a collaborative home-based diabetes intervention to reduce emergency department visits and hospitalizations in black individuals with diabetes. Contemp Clin Trials. 2020;95:106069. 32561466 10.1016/j.cct.2020.106069

[18] **Gounder PP, Seeman SM, Holman RC, et al.** Potentially preventable hospitalizations for acute and chronic conditions in Alaska, 2010-2012. Prev Med Rep. 2016;4:614-21. 27920972 10.1016/j.pmedr.2016.03.017 PMC5129160

[19] **Quinn N, Gupta N.** Income inequalities in the risk of potentially avoidable hospitaliation and readmission for chronic obstructive pulmonary disease: a population data linkage analysis. Int J Popul Data Sci. 2020;5(3):1370-. 34007889 10.23889/ijpds.v5i1.1388 PMC8110890

[20] **Shahu A, Herrin J, Dhruva SS, et al.** Disparities in socioeconomic context and association with blood pressure control and cardiovascular outcomes in ALLHAT. J Am Heart Assoc. 2019;8(15):e012277. 31362591 10.1161/JAHA.119.012277 PMC6761647

[21] **Will JC, Nwaise IA, Schieb L, Zhong Y.** Geographic and racial patterns of preventable hospitalizations for hypertension: Medicare beneficiaries, 2004-2009. Public Health Rep. 2014;129(1):8-18. 24381355 10.1177/003335491412900104 PMC3862999

[22] **Desai R, Singh S, Syed MH, et al.** Temporal trends in the prevalence of diabetes decompensation (diabetic ketoacidosis and hyperosmolar hyperglycemic state) among adult patients hospitalized with diabetes mellitus: a nationwide analysis stratified by age, gender, and race. Cureus. 2019;11(4):e4353-e. 31192058 10.7759/cureus.4353 PMC6550510

[23] **Ferdinand AO, Akinlotan MA, Callaghan T, Towne SD, Bolin JN.** Factors affecting the likelihood of a hospitalization following a diabetes‐related emergency department visit: a regional and urban‐rural analysis. J Diabetes. 2020;12(9):686-96. 32436371 10.1111/1753-0407.13066

[24] **Mkanta WN, Reece MC, Alamri AD, Ezekekwu EU, Potluri A, Chumbler NR.** A 3-state analysis of black-white disparities in diabetes hospitalizations among Medicaid beneficiaries. Health Serv Res Manag Epidemiol. 2018;5:2333392818783513-. 30083574 10.1177/2333392818783513 PMC6069035

[25] **Balla S, Alqahtani F, Alhajji M, Alkhouli M.** Cardiovascular outcomes and rehospitalization rates in homeless patients admitted with acute myocardial infarction. Mayo Clinic Proceedings. 2020;95(4):660-8. 10.1016/j.mayocp.2020.01.013 32200979

[26] **Husaini BA, Mensah GA, Sawyer D, et al.** Race, sex, and age differences in heart failure-related hospitalizations in a southern state: implications for prevention. Circ Heart Fail. 2011;4(2):161-9. 21178017 10.1161/CIRCHEARTFAILURE.110.958306 PMC3070602

[27] **Kim EJ, Kressin NR, Paasche-Orlow MK, et al.** Racial/ethnic disparities among Asian Americans in inpatient acute myocardial infarction mortality in the United States. BMC Health Serv Res 2018;18(1):370-. 29769083 10.1186/s12913-018-3180-0 PMC5956856

[28] **Basu J, Hanchate A, Koroukian S.** Multiple chronic conditions and disparities in 30-day hospital readmissions among nonelderly adults. J Ambul Care Manag. 2018;41(4):262-73. 10.1097/JAC.0000000000000246 29771742

[29] **Mihailoff M, Deb S, Lee JA, Lynn J.** The effects of multiple chronic conditions on adult patient readmissions and hospital finances: a management case study. Inquiry. 2017;54:46958017729597-. 28863719 10.1177/0046958017729597 PMC5798680

[30] **Vogeli C, Shields AE, Lee TA, et al.** Multiple chronic conditions: prevalence, health consequences, and implications for quality, care management, and costs. J Gen Intern Med. 2007;22 Suppl 3(Suppl 3):391-5. 18026807 10.1007/s11606-007-0322-1 PMC2150598

[31] **Wallar LE, De Prophetis E, Rosella LC.** Socioeconomic inequalities in hospitalizations for chronic ambulatory care sensitive conditions: a systematic review of peer-reviewed literature, 1990-2018. Int J Equity Health. 2020;19(1):60. 32366253 10.1186/s12939-020-01160-0 PMC7197160

[32] **Wolff JL, Starfield B, Anderson G.** Prevalence, expenditures, and complications of multiple chronic conditions in the elderly. Arch Intern Med. 2002;162(20):2269-76. 12418941 10.1001/archinte.162.20.2269

[33] **Gaffney A, White A, Hawks L, et al.** High-deductible health plans and healthcare access, use, and financial strain in those with chronic obstructive pulmonary disease. Ann Am Thorac Soc. 2020;17(1):49-56. 31599647 10.1513/AnnalsATS.201905-400OC

[34] **Diaz Del Valle F, Koff PB, Min S-J, et al.** Challenges faced by rural primary care providers when caring for copd patients in the western United States. Chronic Obstr Pulm Dis. 2021;8(3):336-49. 34048644 10.15326/jcopdf.2021.0215 PMC8428598

[35] **Parekh AK, Goodman RA, Gordon C, Koh HK.** Managing multiple chronic conditions: a strategic framework for improving health outcomes and quality of life. Public Health Rep. 2011;126(4):460-71. 21800741 10.1177/003335491112600403 PMC3115206

[36] **Harris JR, Wallace RB.** The Institute of Medicine’s new report on living well with chronic illness. Prev Chronic Dis. 2012;9:E148-E. 22995102 10.5888/pcd9.120126 PMC3475533

[37] U.S. Department of Health and Human Services. Multiple Chronic Conditions: A Strategic Framework - Optimum Health and Quality of Life for Individuals with Multiple Chronic Conditions [Internet]. Washington, DC: U.S. Department of Health and Human Services; 2010. Available from: https://www.hhs.gov/sites/default/files/ash/initiatives/mcc/mcc_framework.pdf. Accessed 21 June 2023.

[38] **Wagner EH, Austin BT, Davis C, Hindmarsh M, Schaefer J, Bonomi A.** Improving chronic illness care: translating evidence into action. Health Aff. 2001;20(6):64-78. 10.1377/hlthaff.20.6.64 11816692

[39] **Arend J, Tsang-Quinn J, Levine C, Thomas D.** The patient-centered medical home: history, components, and review of the evidence. Mt Sinai J Med. 2012;79(4):433-50. 22786733 10.1002/msj.21326

[40] **Goodrich DE, Kilbourne AM, Nord KM, Bauer MS.** Mental health collaborative care and its role in primary care settings. Curr Psychiatry Rep. 2013;15(8):383. 23881714 10.1007/s11920-013-0383-2 PMC3759986

[41] **Gee PM, Greenwood DA, Paterniti DA, Ward D, Miller LMS.** The eHealth enhanced chronic care model: a theory derivation approach. J Med Internet Res. 2015;17(4):e86-e. 25842005 10.2196/jmir.4067 PMC4398883

[42] **Hennessey B, Suter P.** The community-based transitions model. Home Healthc Nurse. 2011;29(4):218-30. 21464664 10.1097/NHH.0b013e318211986d

[43] **Davis SM, Jones A, Jaynes ME, et al.** Designing a multifaceted telehealth intervention for a rural population using a model for developing complex interventions in nursing. BMC Nurs. 2020;19:9-. 32042264 10.1186/s12912-020-0400-9 PMC7001246

[44] **Grant R, Greene D.** The health care home model: primary health care meeting public health goals. Am J Public Health. 2012;102(6):1096-103. 22515874 10.2105/AJPH.2011.300397 PMC3483945

[45] **Enthoven AC.** Integrated delivery systems: the cure for fragmentation. Am J Manag Care. 2009;15(10 Suppl):S284-90. 20088632

[46] **Meyers DJ, Chien AT, Nguyen KH, Li Z, Singer SJ, Rosenthal MB.** Association of team-based primary care with health care utilization and costs among chronically ill patients. JAMA Intern Med. 2019;179(1):54-61. 30476951 10.1001/jamainternmed.2018.5118 PMC6583420

[47] **Knafl KA, Deatrick JA, Gallo AM, Skelton B.** Tracing the use of the family management framework and measure: a scoping review. J Fam Nurs. 2021;27(2):87-106. 33749353 10.1177/1074840721994331 PMC8044632

[48] **Brown RS, Peikes D, Peterson G, Schore J, Razafindrakoto CM.** Six features of medicare coordinated care demonstration programs that cut hospital admissions of high-risk patients. Health Aff. 2012;31(6):1156-66. 10.1377/hlthaff.2012.0393 22665827

[49] **Chin MH, Cook S, Drum ML, et al.** Improving diabetes care in midwest community health centers with the health disparities collaborative. Diabetes Care. 2004;27(1):2-8. 14693957 10.2337/diacare.27.1.2

[50] **Garg T, Polenick CA, Schoenborn N, et al.** Innovative strategies to facilitate patient-centered research in multiple chronic conditions. J Clin Med. 2021;10(10):2112. 34068839 10.3390/jcm10102112 PMC8153595

[51] **Tinetti ME, Naik AD, Dodson JA.** Moving from disease-centered to patient goals-directed care for patients with multiple chronic conditions: Patient value-based care. JAMA Cardiol. 2016;1(1):9-10. 27437646 10.1001/jamacardio.2015.0248 PMC6995667

[52] **Walradt J, Alphs Jackson H.** Adapting to the reimbursement landscape: the future of value-based care. Heart Fail Clin. 2020;16(4):479-87. 32888642 10.1016/j.hfc.2020.06.008

[53] **Warren M.** Defining health in the era of value-based care: the six cs of health and healthcare. Cureus. 2017;9(2):e1046. 28367385 10.7759/cureus.1046 PMC5362276

[54] **Bierman AS, Wang J, O’Malley PG, Moss DK.** Transforming care for people with multiple chronic conditions: agency for Healthcare Research and Quality’s research agenda. Health Serv Res. 2021;56 Suppl 1(Suppl 1):973-9. 34378192 10.1111/1475-6773.13863 PMC8515222

[55] **Munn Z, Peters MDJ, Stern C, Tufanaru C, McArthur A, Aromataris E.** Systematic review or scoping review? Guidance for authors when choosing between a systematic or scoping review approach. BMC Med Res Methodol. 2018;18(1):143. 30453902 10.1186/s12874-018-0611-x PMC6245623

[56] **Tricco AC, Lillie E, Zarin W, et al.** PRISMA extension for scoping reviews (PRISMA-ScR): checklist and explanation. Ann Intern Med. 2018;169(7):467-73. 30178033 10.7326/M18-0850

[57] National Institute on Minority and Health Disparities, National Institutes of Health. Minority health and health disparities: Definitions and parameters. Available at: https://www.nimhd.nih.gov/about/strategic-plan/nih-strategic-plan-definitions-and-parameters.html. Accessed 21 June 2024.

[58] **Wagner EH.** Organizing care for patients with chronic illness revisited. Milbank Q. 2019;97(3):659-64. 31424130 10.1111/1468-0009.12416 PMC6739608

[59] **Chan B, Edwards ST, Srikanth P, et al.** Ambulatory intensive care for medically complex patients at a health care clinic for individuals experiencing homelessness: The SUMMIT randomized clinical trial. JAMA Netw Open. 2023;6(11):e2342012-e. 37948081 10.1001/jamanetworkopen.2023.42012 PMC10638646

[60] **Shah MK, Wyatt LC, Gibbs-Tewary C, Zanowiak JM, Mammen S, Islam N.** A culturally adapted, telehealth, community health worker intervention on blood pressure control among south asian immigrants with type II diabetes: results from the DREAM Atlanta intervention. J Gen Intern Med. 2024;39(4):529-39. 37845588 10.1007/s11606-023-08443-6 PMC10973296

[61] **Felker GM, Sharma A, Mentz RJ, et al.** A randomized controlled trial of mobile health intervention in patients with heart failure and diabetes. J Card Fail. 2022;28(11):1575-83. 35882260 10.1016/j.cardfail.2022.07.048

[62] **Junkins A, Psaros C, Ott C, et al.** Feasibility, acceptability, and preliminary impact of telemedicine-administered cognitive behavioral therapy for adherence and depression among African American women living with HIV in the rural South. J Health Psychol. 2021;26(14):2730-42. 32515245 10.1177/1359105320926526 PMC8012083

[63] **Errichetti KS, Flynn A, Gaitan E, Ramirez MM, Baker M, Xuan Z.** Randomized trial of reverse colocated integrated care on persons with severe, persistent mental illness in Southern Texas. J Gen Intern Med. 2020;35(7):2035-42. 32314132 10.1007/s11606-020-05778-2 PMC7351885

[64] **Steinman L, Xing J, Court B, et al.** Can a home-based collaborative care model reduce health services utilization for older Medicaid beneficiaries living with depression and co-occurring chronic conditions? a quasi-experimental study. Adm Policy Ment Health. 2023;50(5):712-24. 37233831 10.1007/s10488-023-01271-0 PMC11753177

[65] **Germack HD, Leung L, Zhao X, Zhang H, Martsolf GR.** Association of team-based care and continuity of care with hospitalizations for veterans with comorbid mental and physical health conditions. J Gen Intern Med. 2022;37(1):40-8. 34027614 10.1007/s11606-021-06884-5 PMC8739416

[66] **Grove LR, Domino ME, Farley JF, et al.** Medical home effects on enrollees with mental and physical illness. Am J Manag Care. 2020;26(5):218-23. 32436679 10.37765/ajmc.2020.43153

[67] **Schuttner L, Wong ES, Rosland AM, Nelson K, Reddy A.** Association of the patient-centered medical home implementation with chronic disease quality in patients with multimorbidity. J Gen Intern Med. 2020;35(10):2932-8. 32767035 10.1007/s11606-020-06076-7 PMC7572962

[68] **Irwin KE, Park ER, Fields LE, et al.** Bridge: person-centered collaborative care for patients with serious mental illness and cancer. Oncologist. 2019;24(7):901-10. 30696722 10.1634/theoncologist.2018-0488 PMC6656464

[69] **Crits-Christoph P, Gallop R, Noll E, et al.** Impact of a medical home model on costs and utilization among comorbid HIV-positive Medicaid patients. Am J Manag Care. 2018;24(8):368-75. 30130029 PMC6290667

[70] **Gilmer TP, Avery M, Siantz E, et al.** Evaluation of the behavioral health integration and complex care initiative in Medi-Cal. Health Aff. 2018;37(9):1442-9. 10.1377/hlthaff.2018.0372 30179553

[71] **Matzke GR, Moczygemba LR, Williams KJ, Czar MJ, Lee WT.** Impact of a pharmacist-physician collaborative care model on patient outcomes and health services utilization. Am J Health Syst Pharm. 2018;75(14):1039-47. 29789318 10.2146/ajhp170789

[72] **Swietek KE, Domino ME, Beadles C, et al.** Do medical homes improve quality of care for persons with multiple chronic conditions? Health Serv Res. 2018;53(6):4667-81. 30088272 10.1111/1475-6773.13024 PMC6232445

[73] **Taber DJ, Gebregziabher M, Posadas A, Schaffner C, Egede LE, Baliga PK.** Pharmacist-led, technology-assisted study to improve medication safety, cardiovascular risk factor control, and racial disparities in kidney transplant recipients. J Am Coll Clin Pharm. 2018;1(2):81-8. 30714026 10.1002/jac5.1024 PMC6350824

[74] **Rhodes KV, Basseyn S, Gallop R, Noll E, Rothbard A, Crits-Christoph P.** Pennsylvania’s medical home initiative: Reductions in healthcare utilization and cost among Medicaid patients with medical and psychiatric comorbidities. J Gen Intern Med. 2016;31(11):1373-81. 27353455 10.1007/s11606-016-3734-y PMC5071276

[75] **Rossom RC, Solberg LI, Magnan S, et al.** Impact of a national collaborative care initiative for patients with depression and diabetes or cardiovascular disease. Gen Hosp Psychiatry. 2017;44:77-85. 27558106 10.1016/j.genhosppsych.2016.05.006

[76] **Savitz LA, Bayliss EA.** Emerging models of care for individuals with multiple chronic conditions. Health Serv Res. 2021;56(S1):980-9. 34387358 10.1111/1475-6773.13774 PMC8515217

[77] **van Eck van der Sluijs JF, Castelijns H, Eijsbroek V, Rijnders CAT, van Marwijk HWJ, van der Feltz-Cornelis CM.** Illness burden and physical outcomes associated with collaborative care in patients with comorbid depressive disorder in chronic medical conditions: a systematic review and meta-analysis. Gen Hosp Psychiatry. 2018;50:1-14. 28957682 10.1016/j.genhosppsych.2017.08.003

[78] **Banstola A, Pokhrel S, Hayhoe B, Nicholls D, Harris M, Anokye N.** Economic evaluations of interventional opportunities for the management of mental–physical multimorbidity: a systematic review. BMJ Open. 2023;13(2):e069270. 36854591 10.1136/bmjopen-2022-069270 PMC9980364

[79] **Barajas-Nava LA, Garduño-Espinosa J, Mireles Dorantes JM, Medina-Campos R, García-Peña MC.** Models of comprehensive care for older persons with chronic diseases: a systematic review with a focus on effectiveness. BMJ Open. 2022;12(8):e059606. 36170225 10.1136/bmjopen-2021-059606 PMC9362834

[80] **Eriksen CU, Kamstrup-Larsen N, Birke H, et al.** Models of care for improving health-related quality of life, mental health, or mortality in persons with multimorbidity: a systematic review of randomized controlled trials. J Multimorb Comorb. 2022;12:26335565221134017. 36325259 10.1177/26335565221134017 PMC9618762

[81] **Wagner EH, Austin BT, Von Korff M.** Organizing care for patients with chronic illness. Milbank Q. 1996;74(4):511-44. 8941260

[82] National Academies of Sciences Engineering and Medicine. Integrating Social Care into the Delivery of Health Care: Moving Upstream to Improve the Nation’s Health. Washington, DC: National Academies Press; 2019. Available from: https://nap.nationalacademies.org/catalog/25467/integrating-social-care-into-the-delivery-of-health-care-moving. Accessed 3 July 2024. 31940159

[83] **Longhini J, Canzan F, Mezzalira E, Saiani L, Ambrosi E.** Organisational models in primary health care to manage chronic conditions: a scoping review. Health Soc Care Community. 2022;30(3):e565-e88. 10.1111/hsc.13611 34672051

[84] **Ludwick T, Endriyas M, Morgan A, Kane S, Kelaher M, McPake B.** Challenges in implementing community-based healthcare teams in a low-income country context: lessons From Ethiopia’s family health teams. Int J Health Policy Manag. 2022;11(8):1459-71. 34273919 10.34172/ijhpm.2021.52 PMC9808330

[85] **Pinter KA, Zhang H, Liu C, et al.** Elements and performance indicators of integrated healthcare programmes on chronic diseases in six countries in the Asia-Pacific region: a scoping review. Int J Integr Care. 2021;21(1):3. 33613135 10.5334/ijic.5439 PMC7879996

[86] **Wilson MG, Lavis JN, Gauvin FP.** Designing integrated approaches to support people with multimorbidity: key messages from systematic reviews, health system leaders and citizens. Healthc Policy. 2016;12(2):91-104. 28032827 PMC5221714

[87] **Rohwer A, Uwimana Nicol J, Toews I, Young T, Bavuma CM, Meerpohl J.** The effects of integrated models of care for diabetes and hypertension in low-income and middle-income countries: a systematic review and meta-analysis. BMJ Open. 2021;11(7):e043705. 34253658 10.1136/bmjopen-2020-043705 PMC8276295

[88] Institute of Medicine. Living well with chronic illness: A call for public health action [Internet]. Washington, DC: National Academies Press; 2012. Available from: https://nap.nationalacademies.org/catalog/13272/living-well-with-chronic-illness-a-call-for-public-health. Accessed 21 June 2023.

[89] Institute of Medicine (US) Committee on Quality of Health Care in America. Crossing the Quality Chasm: A New Health System for the 21st Century [Internet]. Washington (DC): National Academies Press; 2001. Available from: https://pubmed.ncbi.nlm.nih.gov/25057539/. Accessed 21 June 2023. 25057539

[90] **Ray KN, Chari AV, Engberg J, Bertolet M, Mehrotra A.** Disparities in time spent seeking medical care in the United States. JAMA Intern Med. 2015;175(12):1983-6. 26437386 10.1001/jamainternmed.2015.4468 PMC5055855

[91] **Weaver SJ, Che XX, Petersen LA, Hysong SJ.** Unpacking care coordination through a multiteam system lens: A conceptual framework and systematic review. Med Care. 2018;56(3):247-59. 29356720 10.1097/MLR.0000000000000874

[92] **Koirala B, Peeler A, Dennison Himmelfarb C, Davidson PM.** Living with multiple chronic conditions: how we achieve holistic care and optimize health outcomes. J Adv Nurs. 2023;79(2):e7-e9. 36062872 10.1111/jan.15433 PMC9877113

[93] **Samal L, Fu HN, Camara DS, Wang J, Bierman AS, Dorr DA.** Health information technology to improve care for people with multiple chronic conditions. Health Serv Res. 2021;56 Suppl 1(Suppl 1):1006-36. 34363220 10.1111/1475-6773.13860 PMC8515226

[94] **Kruk ME, Gage AD, Arsenault C, et al.** High-quality health systems in the Sustainable Development Goals era: time for a revolution. Lancet Glob Health. 2018;6(11):e1196-e252. 30196093 10.1016/S2214-109X(18)30386-3 PMC7734391

[95] **Parsons A, Unaka NI, Stewart C, et al.** Seven practices for pursuing equity through learning health systems: notes from the field. Learn Health Syst. 2021;5(3):e10279. 34277945 10.1002/lrh2.10279 PMC8278437

[96] **DeMeester RH, Xu LJ, Nocon RS, Cook SC, Ducas AM, Chin MH.** Solving disparities through payment and delivery system reform: a program to achieve health equity. Health Aff. 2017;36(6):1133-9. 10.1377/hlthaff.2016.0979 28583973

