# Supplementary material for: Health Care Models for Persons with Multiple Chronic Conditions from Populations that Experience Health Disparities: A Scoping Review
Source: J Gen Intern Med. 2025 Apr 23;40(10):2346–57. doi: 10.1007/s11606-025-09491-w (PMC12343438; doi:10.1007/s11606-025-09491-w)
Supplement: Supplementary file 2 — Supplementary file2 (DOCX 31 KB) [file 11606_2025_9491_MOESM2_ESM.docx]

**Appendix 1.** Study inclusion criteria per level of screening

| 1. **Screening Criteria for Title and Abstract Screening** | |
| --- | --- |
| Population: | - Adults 18 years and older residing in the United States - Have a diagnosis of two or more chronic conditions^1^   - At least one of the conditions is not a mental health condition   - Conditions need to be independent and not secondary to the other^2,3^ |
| Indicator: | - Any health care delivery model, intervention, approach, or strategy for improving coordination of comprehensive health care and health outcomes for two or more chronic health conditions   - Must focus on the management of at least 2 chronic conditions |
| Outcome: | - Any process of care (e.g., receipt of guideline concordant care – HbA1c test) or outcome (e.g., HbA1c level, blood pressure, health-related quality of life, mortality, economic evaluations, out of pocket costs/copays) measured at the patient level and related to both chronic conditions studied4 |
| Study types: | - Analytical studies, including randomized controlled, quasi-experimental, cohort, case-control, cross-sectional, and case reports/ series. Studies may use quantitative, qualitative, or mixed methods. Studies without a comparison group are included. |
| Language: | - English |
| 1. **Additional Screening Criteria for Full-text Screening** | |
| Population of focus on health disparities: | - At least half of the study population must include a population experiencing health disparities or be in a health care setting that serves such populations using the National Institutes of Health (NIH) designated populations that experience health disparities^5^:   - Racial and/or ethnic minority groups – American Indian or Alaska Native, Asian, Black or African American, Latino or Hispanic, Native Hawaiian and Pacific Islander   - People with lower socioeconomic status   - Underserved rural communities   - Sexual and gender minority groups   - People with disabilities |

Eligible chronic conditions include obesity, diabetes (types 1 and 2) and its complications, cardiovascular diseases (e.g., coronary artery disease, heart failure, peripheral vascular disease, and stroke), cardiovascular or cardiometabolic disease risk factors (e.g., hypertension, hypercholesterolemia/ dyslipidemia, smoking, prediabetes), all cancers and its complications, chronic respiratory diseases (especially chronic obstructive pulmonary disease and asthma), sleep disordered breathing, cognitive decline (e.g., Alzheimer’s disease and related dementias), chronic liver disease and cirrhosis, chronic kidney disease (e.g., end-stage kidney disease), human immunodeficiency virus, substance use disorders, osteoarthritis, systemic lupus erythematosus and its associated complications, and mental health conditions (e.g., anxiety and depression).

2 By secondary we mean that one of the conditions is a result or consequence of the other condition. When the two conditions are in the same disease pathway, then exclude. When it is unclear, pass on to full-text screening.

3 For example, if the study states that they include people with high blood pressure and diabetes, but it is unclear if people had only one condition (either high blood pressure or diabetes), then pass it on to full-text review to evaluate that participants have both conditions.

^4^ For example, if a study population is on people with depression and cancer, then the study needs to examine outcomes related to depression and cancer. The study would be excluded if the outcome measure only examines depression symptoms in cancer patients without reporting outcomes related to cancer.

^5^ NIH-designated populations that experience health disparities as of 2023 was used for this scoping review, which the Middle Eastern and North African (MENA) population had not been officially designated as a racial and/or ethnic minority group.
