# Supplementary material for: Health Care Models for Persons with Multiple Chronic Conditions from Populations that Experience Health Disparities: A Scoping Review
Source: J Gen Intern Med. 2025 Apr 23;40(10):2346–57. doi: 10.1007/s11606-025-09491-w (PMC12343438; doi:10.1007/s11606-025-09491-w)
Supplement: Supplementary file 3 — Supplementary file3 (DOCX 92 KB) [file 11606_2025_9491_MOESM3_ESM.docx]

Appendix 3. Search Strategies

**Title of Review:** Health Care Models for Persons with Multiple Chronic Conditions from Populations that Experience Health Disparities: A Scoping Review

# Search Strategies Used for Update with Additional Terms

**Database:** PubMed
**Platform:** US National Library of Medicine
**Database Date Coverage:** 1946–present

**Date Searched:** March 28, 2022 / **Updated Search:** February 28, 2024
**Date Limits:** 2016–2023
**Other Limits/Filters:** English; Excludes: letters, editorials, commentaries, conference abstracts, reviews, meta-analyses

| **Set** | **Concept** | **Search Strategy** |
| --- | --- | --- |
| #1 | Healthcare model | ("Delivery of Health Care, Integrated"[Majr:Noexp] OR "integrated care"[Title/Abstract:~3] OR "integrated delivery"[Title/Abstract:~3] OR "vertical integration"[Title/Abstract] OR "virtual integration"[Title/Abstract] OR "physician system integration"[Title/Abstract] OR "functional integration"[Title/Abstract] OR "horizontal integration"[Title/Abstract] OR "clinical integration"[Title/Abstract] OR "managed care program*"[Title/Abstract] OR "patient centred care"[Title/Abstract] OR "integrated medicine"[Title/Abstract] OR "integrated social network"[Title/Abstract] OR "integrated social networks"[Title/Abstract] OR "integration of care"[Title/Abstract] OR "patient group care"[Title/Abstract:~2] OR "group medical care"[Title/Abstract:~2] OR "mobile clinic"[Title/Abstract:~2] OR "mobile clinics"[Title/Abstract:~2] OR "mobile health"[Title/Abstract] OR "Mobile Health Units"[Mesh]) |
| #2 | Multiple chronic conditions | ("chronic condition*"[Title/Abstract] OR "co morbidit*"[Title/Abstract] OR comorbidit*[Title/Abstract] OR “multi-morbidit*”[Title/Abstract] OR multimorbidity*[Title/Abstract] OR "chronic illness*"[Title/Abstract] OR "chronic disease*"[Title/Abstract] OR polymorbidity[Title/Abstract] OR polymorbidities[Title/Abstract] OR “poly morbidity”[Title/Abstract] OR “poly morbidities”[Title/Abstract] OR diabetes[Title/Abstract] OR diabetic*[Title/Abstract] OR prediabet*[Title/Abstract] OR "cardiovascular disease*"[Title/Abstract] OR “cardiometabolic disease*”[Title/Abstract] OR “cardiometabolic condition*”[Title/Abstract] OR “cardiovascular condition*”[Title/Abstract] OR hypertension[Title/Abstract] OR "high blood pressure"[Title/Abstract] OR “coronary artery disease*”[Title/Abstract] OR “heart failure”[Title/Abstract] OR “peripheral vascular disease*”[Title/Abstract] OR “heart disease*”[Title/Abstract] OR “vascular disease*”[Title/Abstract] OR hyperlipidemia[Title/Abstract] OR hypercholesterolemia[Title/Abstract] OR dyslipidemia[Title/Abstract] OR "high cholesterol"[Title/Abstract] OR stroke[Title/Abstract] OR "cerebrovascular accident*"[Title/Abstract] OR “chronic respiratory disease*”[Title/Abstract] OR “chronic obstructive pulmonary disease*”[Title/Abstract] OR COPD[Title/Abstract] OR “chronic obstructive airway disease”[Title/Abstract] OR asthma[Title/Abstract] OR “cognitive decline”[Title/Abstract] OR Alzheimer*[Title/Abstract] OR dementia*[Title/Abstract] OR "chronic kidney disease*"[Title/Abstract] OR "chronic kidney failure"[Title/Abstract] OR “chronic liver disease*”[Title/Abstract] OR “chronic liver failure”[Title/Abstract] OR cirrhosis[Title/Abstract] OR "chronic renal disease*"[Title/Abstract] OR "chronic renal insufficienc*"[Title/Abstract] OR “end stage renal disease*”[Title/Abstract] OR osteoarthritis[Title/Abstract] OR “chronic musculoskeletal disease*”[Title/Abstract] OR “systemic lupus erythematosus”[Title/Abstract] OR "autoimmune disease*"[Title/Abstract] OR "auto immune disease*"[Title/Abstract] OR obesity[Title/Abstract] OR obese[Title/Abstract] OR Obesity[Majr:Noexp] OR cancer[Title/Abstract] OR cancers[Title/Abstract] OR "Chronic Disease"[Majr:Noexp] OR "Multiple Chronic Conditions"[Majr] OR "Stroke"[Majr:Noexp] OR "Diabetes Mellitus, Type 1"[Majr:Noexp] OR "Diabetes Mellitus, Type 2"[Majr:Noexp] OR "Kidney Failure, Chronic"[Majr:Noexp] OR "Renal Insufficiency, Chronic"[Majr:Noexp] OR "Hypertension"[Majr:Noexp] OR "Hyperlipidemias"[Majr:Noexp] OR "Cardiovascular Diseases"[Majr:Noexp] OR "Heart Diseases"[Majr:Noexp] OR "Dyslipidemias"[Majr:Noexp] OR Asthma[Majr:Noexp] OR "Alzheimer Disease"[Majr:Noexp] OR "Dementia"[Majr:Noexp] OR "Osteoarthritis"[Majr:Noexp] OR "Lupus Erythematosus, Systemic"[Majr:Noexp] OR Liver Cirrhosis[Majr:Noexp] OR "Pulmonary Disease, Chronic Obstructive"[Majr:Noexp] OR "Autoimmune Diseases"[Majr:Noexp] OR depression[Title/Abstract] OR “Depressive disorder*”[Title/Abstract] OR “bipolar disorder”[Title/Abstract] OR schizophrenia[Title/Abstract] OR schizophrenic[Title/Abstract] OR “mental disorder*”[Title/Abstract] OR “mental health disorder”[Title/Abstract] OR “mental health disorders”[Title/Abstract] OR "Bipolar Disorder"[Majr] OR "Schizophrenia"[Majr:Noexp] OR "Depression"[Majr] OR "Depressive Disorder"[Majr:Noexp] OR “Mental Disorders”[Majr:Noexp]) |
| #3 | United States | (((Northwest*[Title/Abstract] OR northeast*[Title/Abstract] OR southwest*[Title/Abstract] OR southeast*[Title/Abstract] OR midwest*[Title/Abstract] OR southern[Title/Abstract] OR northern[Title/Abstract] OR "new england"[Title/Abstract] OR "mid atlantic"[Title/Abstract] OR "Great lakes region"[Title/Abstract] OR "appalachian region"[Title/Abstract] OR “pacific northwest”[Title/Abstract]) AND (USA[Title/Abstract] OR "United States"[Title/Abstract] OR US[Title] OR u.s.[Title] OR u.s.a.[Title/Abstract])) OR ((ALABAMA[Title/Abstract] OR ALASKA[Title/Abstract] OR ARIZONA[Title/Abstract] OR ARKANSAS[Title/Abstract] OR CALIFORNIA[Title/Abstract] OR COLORADO[Title/Abstract] OR CONNECTICUT[Title/Abstract] OR DELAWARE[Title/Abstract] OR FLORIDA[Title/Abstract] OR GEORGIA[Title/Abstract] OR HAWAII[Title/Abstract] OR IDAHO[Title/Abstract] OR ILLINOIS[Title/Abstract] OR INDIANA[Title/Abstract] OR IOWA[Title/Abstract] OR KANSAS[Title/Abstract] OR KENTUCKY[Title/Abstract] OR LOUISIANA[Title/Abstract] OR MAINE[Title/Abstract] OR MARYLAND[Title/Abstract] OR MASSACHUSETTS[Title/Abstract] OR MICHIGAN[Title/Abstract] OR MINNESOTA[Title/Abstract] OR MISSISSIPPI[Title/Abstract] OR MISSOURI[Title/Abstract] OR MONTANA[Title/Abstract] OR NEBRASKA[Title/Abstract] OR NEVADA[Title/Abstract] OR "NEW HAMPSHIRE"[Title/Abstract] OR "NEW JERSEY"[Title/Abstract] OR "NEW MEXICO"[Title/Abstract] OR "NEW YORK"[Title/Abstract] OR "NORTH CAROLINA"[Title/Abstract] OR "NORTH DAKOTA"[Title/Abstract] OR OHIO[Title/Abstract] OR OKLAHOMA[Title/Abstract] OR OREGON[Title/Abstract] OR PENNSYLVANIA[Title/Abstract] OR "RHODE ISLAND"[Title/Abstract] OR "SOUTH CAROLINA"[Title/Abstract] OR "SOUTH DAKOTA"[Title/Abstract] OR TENNESSEE[Title/Abstract] OR TEXAS[Title/Abstract] OR UTAH[Title/Abstract] OR VERMONT[Title/Abstract] OR VIRGINIA[Title/Abstract] OR WASHINGTON[Title/Abstract] OR "WEST VIRGINIA"[Title/Abstract] OR WISCONSIN[Title/Abstract] OR WYOMING[Title/Abstract] OR USA[Title/Abstract] OR "United States"[Title/Abstract] OR US[Title] OR u.s.[Title] OR u.s.a.[Title/Abstract] OR American*[Title/Abstract] OR GUAM[Title/Abstract] OR "PUERTO RICO"[Title/Abstract] OR "VIRGIN ISLANDS"[Title/Abstract] OR "American Samoa"[Title/Abstract] OR "Northern Mariana Islands"[Title/Abstract] OR "New York City"[Title/Abstract] OR "Los Angeles"[Title/Abstract] OR Houston[Title/Abstract] OR Dallas[Title/Abstract] OR Miami[Title/Abstract] OR Chicago[Title/Abstract] OR Dallas[Title/Abstract] OR Detroit[Title/Abstract] OR Seattle[Title/Abstract] OR "San Francisco"[Title/Abstract] OR Sacramento[Title/Abstract] OR "San Diego"[Title/Abstract] OR Boston[Title/Abstract] OR "Washington DC"[Title/Abstract] OR “district of columbia”[Title/Abstract] OR Baltimore[Title/Abstract] OR Pittsburgh[Title/Abstract] OR Philadelphia[Title/Abstract] OR Denver[Title/Abstract] OR Phoenix[Title/Abstract] OR "New Orleans"[Title/Abstract] OR ALABAMA[Mesh] OR ALASKA[Mesh] OR ARIZONA[Mesh] OR ARKANSAS[Mesh] OR CALIFORNIA[Mesh] OR COLORADO[Mesh] OR CONNECTICUT[Mesh] OR DELAWARE[Mesh] OR FLORIDA[Mesh] OR GEORGIA[Mesh] OR HAWAII[Mesh] OR IDAHO[Mesh] OR ILLINOIS[Mesh] OR INDIANA[Mesh] OR IOWA[Mesh] OR KANSAS[Mesh] OR KENTUCKY[Mesh] OR LOUISIANA[Mesh] OR MAINE[Mesh] OR MARYLAND[Mesh] OR MASSACHUSETTS[Mesh] OR MICHIGAN[Mesh] OR MINNESOTA[Mesh] OR MISSISSIPPI[Mesh] OR MISSOURI[Mesh] OR MONTANA[Mesh] OR NEBRASKA[Mesh] OR NEVADA[Mesh] OR "NEW HAMPSHIRE"[Mesh] OR "NEW JERSEY"[Mesh] OR "NEW MEXICO"[Mesh] OR "NEW YORK"[Mesh] OR "NORTH CAROLINA"[Mesh] OR "NORTH DAKOTA"[Mesh] OR OHIO[Mesh] OR OKLAHOMA[Mesh] OR OREGON[Mesh] OR PENNSYLVANIA[Mesh] OR "RHODE ISLAND"[Mesh] OR "SOUTH CAROLINA"[Mesh] OR "SOUTH DAKOTA"[Mesh] OR TENNESSEE[Mesh] OR TEXAS[Mesh] OR UTAH[Mesh] OR VERMONT[Mesh] OR VIRGINIA[Mesh] OR WASHINGTON[Mesh] OR "WEST VIRGINIA"[Mesh] OR WISCONSIN[Mesh] OR WYOMING[Mesh] OR "United States"[Mesh] OR "American Samoa"[Mesh] OR GUAM[Mesh] OR "PUERTO RICO"[Mesh] OR "United States VIRGIN ISLANDS"[Mesh] OR "New York City"[Mesh] OR "District of Columbia"[Mesh] OR "Philadelphia"[Mesh] OR "Baltimore"[Mesh] OR Boston[Mesh] OR Chicago[Mesh] OR "Los Angeles"[Mesh] OR "New Orleans"[Mesh] OR "San Francisco"[Mesh] OR Appalachian Region[Mesh] OR Great Lakes Region[Mesh] OR Mid-Atlantic Region[Mesh] OR Midwestern United States[Mesh] OR New England[Mesh] OR Northwestern United States[Mesh] OR Pacific States[Mesh] OR Southeastern United States[Mesh] OR Southwestern United States[Mesh]) NOT dollar*[Title/Abstract])) |
| #4 |  | #1 AND #2 AND #3 |
| #5 | Limit Applied: Publication year | #4 AND ("2016/01/01"[PDAT] : "2023/12/31"[PDAT]) AND English[lang] |
| #6 | Limit Applied: Exclude publication type | #5 NOT (letter[Publication Type] OR editorial[Publication Type] OR comment[Publication Type] OR news[Publication Type] OR "Congress"[Publication Type] OR "Consensus Development Conference"[Publication Type] OR editorial[Title/Abstract] OR commentary[Title/Abstract] OR “conference abstract*”[Title/Abstract] OR “conference proceeding*”[Title/Abstract] OR symposium*[Title/Abstract] OR "Published Erratum"[Publication Type] OR errata[Title/Abstract] OR erratum[Title/Abstract] OR corrigenda[Title/Abstract] OR corrigendum[Title/Abstract] OR protocol[Title] OR protocols[Title] OR “meta-analysis”[Title/Abstract] OR “meta-analyses”[Title/Abstract] OR metanalyses[Title/Abstract] OR metanalysis[Title/Abstract] OR “meta analyses”[Title/Abstract] OR “meta analysis”[Title/Abstract] OR "Review"[Publication Type] OR “systematic review*”[Title/Abstract] OR "Systematic Review"[Publication Type] OR "Meta-Analysis" [Publication Type] OR "Network Meta-Analysis"[Mesh] OR "Review"[Publication Type]) |

**Notes:** The limits for language (English) and publication year (2016–2023) were applied to the main search using the filters available in PubMed. The keywords were searched in the title and abstract fields in PubMed (i.e., [Title/Abstract]), text field (i.e., [Text Word]), title (i.e., [Title]), and the controlled vocabulary terms are indicated with [Mesh]; the Mesh terms were also searched as Major Mesh and explosion turned off (i.e., [Majr:Noexp]). Terms searched in the [Text Word] field are searched in the title, abstract, keywords, and MeSH fields. Proximity searching was used to capture multiple terms appearing in any order within a specified distance of one another in the title and abstract fields (i.e., [Title/Abstract:~4]). Phrases were enclosed in quotation marks to force the searching of the exact terms in order presented. To these results, the search strategy to exclude specific publication types was used. No other limits were applied to the searches.

**Database:** Embase
**Platform:** Elsevier

**Database Date Coverage:** 1947–present
**Date Searched:** March 28, 2022 / **Updated Search:** February 28, 2024
**Date Limits:** 2016–2023
**Other Limits/Filters:** Language: English; Source: Embase; Excludes: letters, editorials, commentaries, conference abstracts, reviews, meta-analyses

| **Set** | **Concept** | **Search Strategy** |
| --- | --- | --- |
| #1 | Healthcare model | ('vertical integration':ti,ab OR 'virtual integration':ti,ab OR 'physician system integration':ti,ab OR 'functional integration':ti,ab OR 'horizontal integration':ti,ab OR 'clinical integration':ti,ab OR 'managed care program*':ti,ab OR 'patient centred care':ti,ab OR 'integrated medicine':ti,ab OR 'integrated social network':ti,ab OR 'integrated social networks':ti,ab OR 'integration of care':ti,ab OR 'mobile health':ti,ab OR (integrated NEAR/3 care):ti,ab OR (integrated NEAR/3 delivery):ti,ab OR (mobile NEAR/2 clinic*):ti,ab OR ('patient group' NEAR/2 care):ti,ab OR (group NEAR/3 'medical care'):ti,ab OR 'integrated health care system'/mj OR 'mobile health'/mj OR 'field hospital'/mj) |
| #2 | Multiple chronic conditions | ('chronic condition*':ti,ab OR 'co morbidit*':ti,ab OR comorbidit*:ti,ab OR 'multi-morbidit*':ti,ab OR multimorbidity*:ti,ab OR 'chronic illness*':ti,ab OR 'chronic disease*':ti,ab OR polymorbidity:ti,ab OR polymorbidities:ti,ab OR 'poly morbidity':ti,ab OR 'poly morbidities':ti,ab OR diabetes:ti,ab OR diabetic*:ti,ab OR prediabet*:ti,ab OR 'cardiovascular disease*':ti,ab OR 'cardiometabolic disease*':ti,ab OR 'cardiometabolic condition*':ti,ab OR 'cardiovascular condition*':ti,ab OR hypertension:ti,ab OR 'high blood pressure':ti,ab OR 'coronary artery disease*':ti,ab OR 'heart failure':ti,ab OR 'peripheral vascular disease*':ti,ab OR 'heart disease*':ti,ab OR 'vascular disease*':ti,ab OR hyperlipidemia:ti,ab OR hypercholesterolemia:ti,ab OR dyslipidemia:ti,ab OR 'high cholesterol':ti,ab OR stroke:ti,ab OR 'cerebrovascular accident*':ti,ab OR 'chronic respiratory disease*':ti,ab OR 'chronic obstructive pulmonary disease*':ti,ab OR COPD:ti,ab OR 'chronic obstructive airway disease':ti,ab OR asthma:ti,ab OR 'cognitive decline':ti,ab OR Alzheimer*:ti,ab OR dementia*:ti,ab OR 'chronic kidney disease*':ti,ab OR 'chronic kidney failure':ti,ab OR 'chronic liver disease*':ti,ab OR 'chronic liver failure':ti,ab OR cirrhosis:ti,ab OR 'chronic renal disease*':ti,ab OR 'chronic renal insufficienc*':ti,ab OR 'end stage renal disease*':ti,ab OR osteoarthritis:ti,ab OR 'chronic musculoskeletal disease*':ti,ab OR 'systemic lupus erythematosus':ti,ab OR 'autoimmune disease*':ti,ab OR 'auto immune disease*':ti,ab OR obesity:ti,ab OR obese:ti,ab OR cancer:ti,ab OR cancers:ti,ab OR depression:ti,ab OR 'depressive disorder*':ti,ab OR 'bipolar disorder':ti,ab OR schizophrenia:ti,ab OR schizophrenic:ti,ab OR 'mental disorder*':ti,ab OR 'mental health disorder':ti,ab OR 'mental health disorders':ti,ab OR 'insulin dependent diabetes mellitus'/mj OR 'non insulin dependent diabetes mellitus'/mj OR 'diabetes mellitus'/mj OR 'obesity'/mj OR 'cerebrovascular accident'/mj OR 'hypertension'/mj OR 'hyperlipidemia'/mj OR 'dyslipidemia'/mj OR 'chronic kidney failure'/mj OR 'cardiovascular disease'/mj OR 'heart disease'/mj OR 'asthma'/mj OR 'dementia'/mj OR 'Alzheimer disease'/mj OR 'osteoarthritis'/mj OR 'liver cirrhosis'/mj OR 'chronic obstructive lung disease'/mj OR 'autoimmune disease'/mj OR 'systemic lupus erythematosus'/mj OR 'bipolar disorder'/mj OR 'schizophrenia'/mj OR 'mental disease'/mj OR 'depression'/mj OR 'multiple chronic conditions'/exp OR 'comorbidity'/mj OR 'chronic disease'/exp/mj) |
| #3 | United States | (((Northwest*:ti,ab OR northeast*:ti,ab OR southwest*:ti,ab OR southeast*:ti,ab OR midwest*:ti,ab OR southern:ti,ab OR northern:ti,ab OR 'new england':ti,ab OR 'mid atlantic':ti,ab OR 'Great lakes region':ti,ab OR 'pacific northwest':ti,ab OR 'appalachian region':ti,ab) AND (USA:ti,ab OR 'United States':ti,ab OR US:ti OR u.s.:ti OR u.s.a.:ti,ab)) OR ((ALABAMA:ti,ab OR ALASKA:ti,ab OR ARIZONA:ti,ab OR ARKANSAS:ti,ab OR CALIFORNIA:ti,ab OR COLORADO:ti,ab OR CONNECTICUT:ti,ab OR DELAWARE:ti,ab OR FLORIDA:ti,ab OR GEORGIA:ti,ab OR HAWAII:ti,ab OR IDAHO:ti,ab OR ILLINOIS:ti,ab OR INDIANA:ti,ab OR IOWA:ti,ab OR KANSAS:ti,ab OR KENTUCKY:ti,ab OR LOUISIANA:ti,ab OR MAINE:ti,ab OR MARYLAND:ti,ab OR MASSACHUSETTS:ti,ab OR MICHIGAN:ti,ab OR MINNESOTA:ti,ab OR MISSISSIPPI:ti,ab OR MISSOURI:ti,ab OR MONTANA:ti,ab OR NEBRASKA:ti,ab OR NEVADA:ti,ab OR 'NEW HAMPSHIRE':ti,ab OR 'NEW JERSEY':ti,ab OR 'NEW MEXICO':ti,ab OR 'NEW YORK':ti,ab OR 'NORTH CAROLINA':ti,ab OR 'NORTH DAKOTA':ti,ab OR OHIO:ti,ab OR OKLAHOMA:ti,ab OR OREGON:ti,ab OR PENNSYLVANIA:ti,ab OR 'RHODE ISLAND':ti,ab OR 'SOUTH CAROLINA':ti,ab OR 'SOUTH DAKOTA':ti,ab OR TENNESSEE:ti,ab OR TEXAS:ti,ab OR UTAH:ti,ab OR VERMONT:ti,ab OR VIRGINIA:ti,ab OR WASHINGTON:ti,ab OR 'WEST VIRGINIA':ti,ab OR WISCONSIN:ti,ab OR WYOMING:ti,ab OR USA:ti,ab OR 'United States':ti,ab OR US:ti OR u.s.:ti OR u.s.a.:ti,ab OR American*:ti,ab OR GUAM:ti,ab OR 'PUERTO RICO':ti,ab OR 'VIRGIN ISLANDS':ti,ab OR 'American Samoa':ti,ab OR 'Northern Mariana Islands':ti,ab OR 'New York City':ti,ab OR 'Los Angeles':ti,ab OR Houston:ti,ab OR Dallas:ti,ab OR Miami:ti,ab OR Chicago:ti,ab OR Dallas:ti,ab OR Detroit:ti,ab OR Seattle:ti,ab OR 'San Francisco':ti,ab OR Sacramento:ti,ab OR 'San Diego':ti,ab OR Boston:ti,ab OR 'Washington DC':ti,ab OR Baltimore:ti,ab OR Pittsburgh:ti,ab OR Philadelphia:ti,ab OR Denver:ti,ab OR Phoenix:ti,ab OR 'New Orleans':ti,ab OR ALABAMA/exp OR ALASKA/exp OR ARIZONA/exp OR ARKANSAS/exp OR CALIFORNIA/exp OR COLORADO/exp OR CONNECTICUT/exp OR DELAWARE/exp OR FLORIDA/exp OR GEORGIA/exp OR HAWAII/exp OR IDAHO/exp OR ILLINOIS/exp OR INDIANA/exp OR IOWA/exp OR KANSAS/exp OR KENTUCKY/exp OR LOUISIANA/exp OR MAINE/exp OR MARYLAND/exp OR MASSACHUSETTS/exp OR MICHIGAN/exp OR MINNESOTA/exp OR MISSISSIPPI/exp OR MISSOURI/exp OR MONTANA/exp OR NEBRASKA/exp OR NEVADA/exp OR 'NEW HAMPSHIRE'/exp OR 'NEW JERSEY'/exp OR 'NEW MEXICO'/exp OR 'NEW YORK'/exp OR 'NORTH CAROLINA'/exp OR 'NORTH DAKOTA'/exp OR OHIO/exp OR OKLAHOMA/exp OR OREGON/exp OR PENNSYLVANIA/exp OR 'RHODE ISLAND'/exp OR 'SOUTH CAROLINA'/exp OR 'SOUTH DAKOTA'/exp OR TENNESSEE/exp OR TEXAS/exp OR UTAH/exp OR VERMONT/exp OR VIRGINIA/exp OR WASHINGTON/exp OR 'WEST VIRGINIA'/exp OR WISCONSIN/exp OR WYOMING/exp OR 'United States'/exp OR 'American Samoa'/exp OR GUAM/exp OR 'PUERTO RICO'/exp OR 'United states VIRGIN ISLANDS'/exp OR 'New York City'/exp OR 'District of Columbia'/exp OR 'Philadelphia'/exp OR 'Baltimore'/exp OR Boston/exp OR Chicago/exp OR 'Los Angeles'/exp OR 'New Orleans'/exp OR 'San Francisco'/exp OR ‘Appalachian Region’:ti,ab OR ‘Great Lakes Region’:ti,ab OR ‘Mid-Atlantic Region’:ti,ab OR ‘Midwestern United States’:ti,ab OR ‘New England’:ti,ab OR ‘Northwestern United States’:ti,ab OR ‘Pacific States’:ti,ab OR ‘Southeastern United States’:ti,ab OR ‘Southwestern United States’:ti,ab) NOT dollar*:ti,ab)) |
| #4 |  | #1 AND #2 AND #3 |
| #5 | Limits Applied: Source, Language, Publication year | #4 AND [embase]/lim AND [english]/lim AND [2016-2023]/py |
| #6 | Limit Applied: Exclude Publication type | #5 NOT ([conference abstract]/lim OR [conference paper]/lim OR [conference review]/lim OR [data papers]/lim OR [editorial]/lim OR [erratum]/lim OR [letter]/lim OR [note]/lim OR [short survey]/lim OR 'conference abstract'/exp OR 'conference paper'/exp OR 'data paper'/exp OR 'editorial'/exp OR 'letter'/exp OR 'erratum'/exp OR 'note'/exp OR 'short survey'/exp OR corrigenda:ti,ab OR corrigendum:ti,ab OR erratum:ti,ab OR errata:ti,ab OR protocol*:ti,ab OR ‘conference abstract*’:ti,ab OR ‘conference proceeding*’:ti,ab OR symposium*:ti,ab OR letter:ti,ab OR editorial:ti,ab OR commentary:ti,ab OR review:ti,ab OR [systematic review]/lim OR 'review'/exp OR 'systematic review'/exp OR [review]/lim OR 'meta analysis':ti,ab OR 'meta analyses':ti,ab OR 'meta-analysis':ti,ab OR 'meta-analyses':ti,ab OR metanalysis:ti,ab OR metanalyses:ti,ab) |

**Notes:** The limits for source (Embase), language (English), and publication year (2016–2023) were applied to the main search using the filters available in Embase. The keywords were searched in the title and abstract fields (i.e., :ti,ab), title (i.e., :ti), and the controlled vocabulary terms are indicated with /mj or /exp; the EMTREE terms were also searched as Major (i.e., /mj) and explosion turned off (i.e., /exp). Proximity searching was used to capture multiple terms appearing in any order within a specified distance of one another in the title and abstract fields (i.e., NEAR/3). Phrases were enclosed in quotation marks to force the searching of the exact terms in order presented. To these results, the search strategy to exclude specific publication types was used. No other limits were applied to the searches.

**Database:** Scopus
**Platform:** Elsevier
**Database Date Coverage:** 1788–present

**Date Searched:** March 28, 2022 / **Updated Search:** February 28, 2024
**Date Limits:** 2016–2023
**Other Limits/Filters:** Language: English; Excludes: letters, editorials, commentaries, conference abstracts, reviews, meta-analyses

| **Set** | **Concept** | **Search Strategy** |
| --- | --- | --- |
| #1 | Healthcare model | Title-Abs-Key(("vertical integration" OR "virtual integration" OR "physician system integration" OR "functional integration" OR "horizontal integration" OR "clinical integration" OR "managed care program*" OR "patient centred care" OR "integrated medicine" OR "integrated social network" OR "integrated social networks" OR "integration of care" OR "mobile health" OR (integrated W/3 care) OR (integrated W/3 delivery) OR (mobile W/2 clinic) OR (mobile W/2 clinics) OR ("patient group" W/2 care) OR (group W/3 "medical care") OR "integrated health care system")) |
| #2 | Multiple chronic conditions | Title-Abs-Key(({chronic condition*} OR {co morbidit*} OR comorbidit* OR {multi-morbidit*} OR multimorbidity* OR {chronic illness*} OR {chronic disease*} OR polymorbidity OR polymorbidities OR {poly morbidity} OR {poly morbidities} OR diabetes OR diabetic* OR prediabet* OR {cardiovascular disease*} OR {cardiometabolic disease*} OR {cardiometabolic condition*} OR {cardiovascular condition*} OR hypertension OR {high blood pressure} OR {coronary artery disease*} OR {heart failure} OR {peripheral vascular disease*} OR {heart disease*} OR {vascular disease*} OR hyperlipidemia OR hypercholesterolemia OR dyslipidemia OR {high cholesterol} OR stroke OR {cerebrovascular accident*} OR {chronic respiratory disease*} OR {chronic obstructive pulmonary disease*} OR COPD OR {chronic obstructive airway disease} OR asthma OR {cognitive decline} OR Alzheimer* OR dementia* OR {chronic kidney disease*} OR {chronic kidney failure} OR {chronic liver disease*} OR {chronic liver failure} OR cirrhosis OR {chronic renal disease*} OR {chronic renal insufficienc*} OR {end stage renal disease*} OR osteoarthritis OR {chronic musculoskeletal disease*} OR {systemic lupus erythematosus} OR {autoimmune disease*} OR {auto immune disease*} OR obesity OR obese OR cancer OR cancers OR depression OR {Depressive disorder*} OR {bipolar disorder} OR schizophrenia OR schizophrenic OR {mental disorder*} OR {mental health disorder} OR {mental health disorders})) |
| #3 | United States | Title-Abs-Key((((Northwest* OR northeast* OR southwest* OR southeast* OR midwest* OR southern OR northern OR {new england} OR {mid atlantic} OR {Great lakes region} OR {appalachian region}) AND (USA OR {United States} OR US OR u.s. OR u.s.a.)) OR ((ALABAMA OR ALASKA OR ARIZONA OR ARKANSAS OR CALIFORNIA OR COLORADO OR CONNECTICUT OR DELAWARE OR FLORIDA OR GEORGIA OR HAWAII OR IDAHO OR ILLINOIS OR INDIANA OR IOWA OR KANSAS OR KENTUCKY OR LOUISIANA OR MAINE OR MARYLAND OR MASSACHUSETTS OR MICHIGAN OR MINNESOTA OR MISSISSIPPI OR MISSOURI OR MONTANA OR NEBRASKA OR NEVADA OR {NEW HAMPSHIRE} OR {NEW JERSEY} OR {NEW MEXICO} OR {NEW YORK} OR {NORTH CAROLINA} OR {NORTH DAKOTA} OR OHIO OR OKLAHOMA OR OREGON OR PENNSYLVANIA OR {RHODE ISLAND} OR {SOUTH CAROLINA} OR {SOUTH DAKOTA} OR TENNESSEE OR TEXAS OR UTAH OR VERMONT OR VIRGINIA OR WASHINGTON OR {WEST VIRGINIA} OR WISCONSIN OR WYOMING OR USA OR {United States} OR US OR u.s. OR u.s.a. OR American* OR GUAM OR {PUERTO RICO} OR {VIRGIN ISLANDS} OR {American Samoa} OR {Northern Mariana Islands} OR {New York City} OR {Los Angeles} OR Houston OR Dallas OR Miami OR Chicago OR Dallas OR Detroit OR Seattle OR {San Francisco} OR Sacramento OR {San Diego} OR Boston OR {Washington DC} OR Baltimore OR Pittsburgh OR Philadelphia OR Denver OR Phoenix OR {New Orleans} OR {Appalachian Region} OR {Great Lakes Region} OR {Mid-Atlantic Region} OR {Midwestern United States} OR {New England} OR {Northwestern United States} OR {Pacific States} OR {Southeastern United States} OR {Southwestern United States}) AND NOT dollar*) )) |
| #4 |  | #1 AND #2 AND #3 |
| #5 | Limits Applied: Language, Publication year | #4 AND ( LIMIT-TO ( PUBYEAR,2023) OR LIMIT-TO ( PUBYEAR,2022) OR LIMIT-TO ( PUBYEAR,2021) OR LIMIT-TO ( PUBYEAR,2020) OR LIMIT-TO ( PUBYEAR,2019) OR LIMIT-TO ( PUBYEAR,2018) OR LIMIT-TO ( PUBYEAR,2017) OR LIMIT-TO ( PUBYEAR,2016) ) AND ( LIMIT-TO ( LANGUAGE,"English" ) ) |
| #6 | Limit Applied: Exclude Publication type | #5 NOT ( EXCLUDE ( DOCTYPE , "re" ) OR EXCLUDE ( DOCTYPE , "no" ) OR EXCLUDE ( DOCTYPE , "ch" ) OR EXCLUDE ( DOCTYPE , "ed" ) OR EXCLUDE ( DOCTYPE , "er" ) OR EXCLUDE ( DOCTYPE , "le" ) ) AND ( LIMIT-TO ( DOCTYPE , "ar" ) ) |

**Notes:** The limits for language (English) and publication year (2016–2023) were applied to the main search using the filters available in Scopus. The keywords were searched in the title, abstract and keywords fields (i.e., Title-Abs-Key). Proximity searching was used to capture multiple terms appearing in any order within a specified distance of one another in the title and abstract fields (i.e., W/3). Phrases were enclosed in quotation marks to force the searching of the exact terms in order presented. To these results, the search strategy to exclude specific publication types was used. No other limits were applied to the searches.

**Database:** CINAHL Plus
**Platform:** EBSCOhost
**Database Date Coverage:** 1937–present

**Date Searched:** March 28, 2022 / **Updated Search:** February 28, 2024
**Date Limits:** 2016–2023
**Other Limits/Filters:** Language: English; Excludes: letters, editorials, commentaries, conference abstracts, reviews, meta-analyses

| **Set** | **Concept** | **Search Strategy** |
| --- | --- | --- |
| #1 | Healthcare model | Title: ("vertical integration" OR "virtual integration" OR "physician system integration" OR "functional integration" OR "horizontal integration" OR "clinical integration" OR "managed care program*" OR "patient centred care" OR "integrated medicine" OR "integrated social network" OR "integrated social networks" OR "integration of care" OR "mobile health" OR "integrated health care system" OR (integrated W3 care) OR (integrated W3 delivery) OR (mobile W2 clinic) OR (mobile W2 clinics) OR ("patient group" W2 care) OR (group W3 "medical care") ) |
| #2 | Healthcare model | Title: ("vertical integration" OR "virtual integration" OR "physician system integration" OR "functional integration" OR "horizontal integration" OR "clinical integration" OR "managed care program*" OR "patient centred care" OR "integrated medicine" OR "integrated social network" OR "integrated social networks" OR "integration of care" OR "mobile health" OR "integrated health care system" OR (integrated W3 care) OR (integrated W3 delivery) OR (mobile W2 clinic) OR (mobile W2 clinics) OR ("patient group" W2 care) OR (group W3 "medical care") ) |
| #3 | Healthcare model | Exact Major Subject Heading: ((MM "Mobile Health Units") OR (MM "Health Care Delivery, Integrated")) |
| #4 | Healthcare model | #1 OR #2 OR #3 |
| #5 | Multiple chronic conditions | Title: ("chronic condition*" OR "co morbidit*" OR comorbidit* OR "multi-morbidit*" OR multimorbidity* OR "chronic illness*" OR "chronic disease*" OR polymorbidity OR polymorbidities OR "poly morbidity" OR "poly morbidities" OR diabetes OR diabetic* OR prediabet* OR "cardiovascular disease*" OR "cardiometabolic disease*" OR "cardiometabolic condition*" OR "cardiovascular condition*" OR hypertension OR "high blood pressure" OR "coronary artery disease*" OR "heart failure" OR "peripheral vascular disease*" OR "heart disease*" OR "vascular disease*" OR hyperlipidemia OR hypercholesterolemia OR dyslipidemia OR "high cholesterol" OR stroke OR "cerebrovascular accident*" OR "chronic respiratory disease*" OR "chronic obstructive pulmonary disease*" OR COPD OR "chronic obstructive airway disease" OR asthma OR "cognitive decline" OR Alzheimer* OR dementia* OR "chronic kidney disease*" OR "chronic kidney failure" OR "chronic liver disease*" OR "chronic liver failure" OR cirrhosis OR "chronic renal disease*" OR "chronic renal insufficienc*" OR "end stage renal disease*" OR osteoarthritis OR "chronic musculoskeletal disease*" OR "systemic lupus erythematosus" OR "autoimmune disease*" OR "auto immune disease*" OR obesity OR obese OR cancer OR cancers OR depression OR "Depressive disorder*" OR "bipolar disorder" OR schizophrenia OR schizophrenic OR "mental disorder*" OR "mental health disorder" OR "mental health disorders") |
| #6 | Multiple chronic conditions | Abstract: ("chronic condition*" OR "co morbidit*" OR comorbidit* OR "multi-morbidit*" OR multimorbidity* OR "chronic illness*" OR "chronic disease*" OR polymorbidity OR polymorbidities OR "poly morbidity" OR "poly morbidities" OR diabetes OR diabetic* OR prediabet* OR "cardiovascular disease*" OR "cardiometabolic disease*" OR "cardiometabolic condition*" OR "cardiovascular condition*" OR hypertension OR "high blood pressure" OR "coronary artery disease*" OR "heart failure" OR "peripheral vascular disease*" OR "heart disease*" OR "vascular disease*" OR hyperlipidemia OR hypercholesterolemia OR dyslipidemia OR "high cholesterol" OR stroke OR "cerebrovascular accident*" OR "chronic respiratory disease*" OR "chronic obstructive pulmonary disease*" OR COPD OR "chronic obstructive airway disease" OR asthma OR "cognitive decline" OR Alzheimer* OR dementia* OR "chronic kidney disease*" OR "chronic kidney failure" OR "chronic liver disease*" OR "chronic liver failure" OR cirrhosis OR "chronic renal disease*" OR "chronic renal insufficienc*" OR "end stage renal disease*" OR osteoarthritis OR "chronic musculoskeletal disease*" OR "systemic lupus erythematosus" OR "autoimmune disease*" OR "auto immune disease*" OR obesity OR obese OR cancer OR cancers OR depression OR "Depressive disorder*" OR "bipolar disorder" OR schizophrenia OR schizophrenic OR "mental disorder*" OR "mental health disorder" OR "mental health disorders") |
| #7 | Multiple chronic conditions | Exact Major Subject Heading: ((MM "Obesity") OR (MM "Chronic Disease") OR (MM "Hypertension") OR (MM "Hyperlipidemia") OR (MM "Hypercholesterolemia") OR (MM "Diabetes Mellitus, Type 1") OR (MM "Diabetes Mellitus, Type 2") OR (MM "Stroke") OR (MM "Kidney Failure, Chronic") OR (MM "Renal Insufficiency, Chronic") OR (MM "Heart Diseases") OR (MM "Cardiovascular Diseases") OR (MM "Schizophrenia") OR (MM "Mental Disorders") OR (MM "Depression") OR (MM "Lupus Erythematosus, Systemic") OR (MM "Autoimmune Diseases") OR (MM "Pulmonary Disease, Chronic Obstructive") OR (MM "Asthma") OR (MM "Liver Cirrhosis") OR (MM "Osteoarthritis") OR (MM "Alzheimer's Disease") OR (MM "Dementia") OR (MM "Comorbidity")) |
| #8 | Multiple chronic conditions | #5 OR #6 OR #7 |
| #9 | United States | Title: (ALABAMA OR ALASKA OR ARIZONA OR ARKANSAS OR CALIFORNIA OR COLORADO OR CONNECTICUT OR DELAWARE OR FLORIDA OR GEORGIA OR HAWAII OR IDAHO OR ILLINOIS OR INDIANA OR IOWA OR KANSAS OR KENTUCKY OR LOUISIANA OR MAINE OR MARYLAND OR MASSACHUSETTS OR MICHIGAN OR MINNESOTA OR MISSISSIPPI OR MISSOURI OR MONTANA OR NEBRASKA OR NEVADA OR "NEW HAMPSHIRE" OR "NEW JERSEY" OR "NEW MEXICO" OR "NEW YORK" OR "NORTH CAROLINA" OR "NORTH DAKOTA" OR OHIO OR OKLAHOMA OR OREGON OR PENNSYLVANIA OR "RHODE ISLAND" OR "SOUTH CAROLINA" OR "SOUTH DAKOTA" OR TENNESSEE OR TEXAS OR UTAH OR VERMONT OR VIRGINIA OR WASHINGTON OR "WEST VIRGINIA" OR WISCONSIN OR WYOMING OR USA OR "United States" OR US OR u.s. OR u.s.a. OR American* OR GUAM OR "PUERTO RICO" OR "VIRGIN ISLANDS" OR "American Samoa" OR "Northern Mariana Islands") |
| #10 | United States | Abstract: (ALABAMA OR ALASKA OR ARIZONA OR ARKANSAS OR CALIFORNIA OR COLORADO OR CONNECTICUT OR DELAWARE OR FLORIDA OR GEORGIA OR HAWAII OR IDAHO OR ILLINOIS OR INDIANA OR IOWA OR KANSAS OR KENTUCKY OR LOUISIANA OR MAINE OR MARYLAND OR MASSACHUSETTS OR MICHIGAN OR MINNESOTA OR MISSISSIPPI OR MISSOURI OR MONTANA OR NEBRASKA OR NEVADA OR "NEW HAMPSHIRE" OR "NEW JERSEY" OR "NEW MEXICO" OR "NEW YORK" OR "NORTH CAROLINA" OR "NORTH DAKOTA" OR OHIO OR OKLAHOMA OR OREGON OR PENNSYLVANIA OR "RHODE ISLAND" OR "SOUTH CAROLINA" OR "SOUTH DAKOTA" OR TENNESSEE OR TEXAS OR UTAH OR VERMONT OR VIRGINIA OR WASHINGTON OR "WEST VIRGINIA" OR WISCONSIN OR WYOMING OR USA OR "United States" OR US OR u.s. OR u.s.a. OR American* OR GUAM OR "PUERTO RICO" OR "VIRGIN ISLANDS" OR "American Samoa" OR "Northern Mariana Islands") |
| #11 | United States | Title: ("New York City" OR "Los Angeles" OR Houston OR Dallas OR Miami OR Chicago OR Dallas OR Detroit OR Seattle OR "San Francisco" OR Sacramento OR "San Diego" OR Boston OR "Washington DC" OR Baltimore OR Pittsburgh OR Philadelphia OR Denver OR Phoenix OR "New Orleans" OR "Appalachian Region" OR "Great Lakes Region" OR "Mid-Atlantic Region" OR "Midwestern United States" OR "New England" OR "Northwestern United States" OR "Pacific States" OR “Pacific Northwest” OR "Southeastern United States" OR "Southwestern United States") |
| #12 | United States | Abstract: ("New York City" OR "Los Angeles" OR Houston OR Dallas OR Miami OR Chicago OR Dallas OR Detroit OR Seattle OR "San Francisco" OR Sacramento OR "San Diego" OR Boston OR "Washington DC" OR Baltimore OR Pittsburgh OR Philadelphia OR Denver OR Phoenix OR "New Orleans" OR "Appalachian Region" OR "Great Lakes Region" OR "Mid-Atlantic Region" OR "Midwestern United States" OR "New England" OR "Northwestern United States" OR "Pacific States" OR “Pacific Northwest” OR "Southeastern United States" OR "Southwestern United States") |
| #13 | United States | Exact Subject Heading: (MH "United States+") OR (MH "United States by Individual State+") OR (MH "United States by Region+") |
| #14 | United States | Title: ((Northwest* OR northeast* OR southwest* OR southeast* OR midwest* OR southern OR northern OR "new england" OR "mid atlantic" OR "Great lakes region" OR "appalachian region" OR “Pacific Northwest”) AND (USA OR "United States" OR US OR u.s. OR u.s.a.)) |
| #15 | United States | Abstract: ((Northwest* OR northeast* OR southwest* OR southeast* OR midwest* OR southern OR northern OR "new england" OR "mid atlantic" OR "Great lakes region" OR "appalachian region" OR “Pacific Northwest”) AND (USA OR "United States" OR US OR u.s. OR u.s.a.)) |
| #16 | United States | #9 OR #10 OR #11 OR #12 OR #13 OR #14 OR #15 OR #16 |
| #17 |  | #4 AND #8 AND #16 |
| #18 | Limits Applied: Publication year, Language, Publication type | #17 AND Publication Year: 2016-2023 AND Language: English AND Publication Type: Journal Article |
| #19 | Limit Applied: Exclude Publication type | Exact Subject Heading: ((MH "News" OR (MH "Theses and Dissertations") OR (MH "Protocols") OR (MH "Systematic Review") OR (MH "Scoping Review") OR (MH "Meta Analysis") OR (MH "Congresses and Conferences")) |
| #20 | Limit Applied: Exclude Publication type | Title: (corrigenda OR corrigendum OR erratum OR errata OR “conference abstract” OR “conference abstracts” OR “conference proceeding” OR “conference proceedings” OR symposium* OR editorial OR commentary OR protocol OR protocols OR “systematic review*” OR “meta analysis” OR “meta analyses” OR metanalysis OR metanalyses) |
| #21 | Limit Applied: Exclude Publication type | Abstract: (corrigenda OR corrigendum OR erratum OR errata OR “conference abstract” OR “conference abstracts” OR “conference proceeding” OR “conference proceedings” OR symposium* OR editorial OR commentary OR protocol OR protocols OR “systematic review*” OR “meta analysis” OR “meta analyses” OR metanalysis OR metanalyses) |
| #22 |  | #19 OR #20 OR #21 |
| #23 | Limit Applied: Exclude Publication type | #18 NOT #22 |

**Notes:** The limits for language (English) and publication year (2016–2023) were applied to the main search using the filters available in CINAHL Plus. The keywords were searched in the title and abstract fields and the controlled vocabulary terms are indicated Exact Subject Headings (MH) or Exact Major Subject Headings (MM). Proximity searching was used to capture multiple terms appearing in any order within a specified distance of one another in the title and abstract fields (i.e., W3). Phrases were enclosed in quotation marks to force the searching of the exact terms in order presented. To these results, the search strategy to exclude specific publication types as specified in the exclusion criteria was used, and the Publication Type limit “Journal Article” was applied.

# Search Strategies Used for Original Search

**Database:** PubMed
**Platform:** US National Library of Medicine
**Database Date Coverage:** 1946–present

**Date Searched:** March 28, 2022
**Date Limits:** 2016–2021
**Other Limits/Filters:** Language: English; Excludes: letters, editorials, commentaries, conference abstracts, reviews, meta-analyses

| **Set** | **Concept** | **Search Strategy** |
| --- | --- | --- |
| #1 | Healthcare model | ("healthcare model*"[Title/Abstract] OR "health care model*"[Title/Abstract] OR “healthcare delivery model*”[Title/Abstract] OR “health care delivery model*”[Title/Abstract] OR “delivery model*”[Title/Abstract] OR “delivery care model*”[Title/Abstract] OR “care model”[Title/Abstract] OR “care models”[Title/Abstract] OR “care delivery model*”[Title/Abstract] OR “multispecialty clinic*”[Title/Abstract] OR “multi-specialty clinic*”[Title/Abstract] OR "integrated care"[Title/Abstract] OR "integrated delivery system*"[Title/Abstract] OR “integrated delivery of healthcare”[Title/Abstract] OR “integrated delivery of health care”[Title/Abstract] OR “integrated practice”[Title/Abstract] OR "community health plan*"[Title/Abstract] OR "value based care"[Title/Abstract] OR "value based healthcare"[Title/Abstract] OR "value based health care"[Title/Abstract] OR “value based payment*”[Title/Abstract] OR “Nurse Care Model*”[Title/Abstract] OR “nurse led”[Title/Abstract] OR "nurse management model*"[Title/Abstract] OR “Chronic Illness Self-management”[Title/Abstract] OR “chronic condition self management”[Title/Abstract] OR “chronic disease self management”[Title/Abstract] OR "Community Health Planning"[Majr] OR "chronic care model*"[Title/Abstract] OR “chronic care initiative*”[Title/Abstract] OR "community based transition*"[Title/Abstract] OR "home based model*"[Title/Abstract] OR “home based care”[Title/Abstract] OR "Patient-Centered Care"[Majr:Noexp] OR "patient centered care"[Title/Abstract] OR “patient centered model*”[Title/Abstract] OR "patient centered medical home*"[Title/Abstract] OR "accountable care organization*"[Title/Abstract] OR "Accountable Care Organizations"[Majr] OR "managed care model*"[Title/Abstract] OR “Delivery of Health Care, Integrated”[Majr:Noexp] OR “Patient Care Management”[Majr:Noexp] OR "Patient Care Planning"[Majr:Noexp] OR “Comprehensive Health Care”[Majr:Noexp] OR “sub specialty care”[Title/Abstract] OR “subspecialty care”[Title/Abstract] OR Telemedicine[Majr:Noexp] OR telemedicine[Title/Abstract] OR telehealth[Title/Abstract] OR “care coordination”[Title/Abstract] OR “coordinated care”[Title/Abstract] OR “multidisciplinary care”[Title/Abstract] OR “multi disciplinary care”[Title/Abstract] OR “multidisciplinary specialist care”[Title/Abstract] OR “multidisciplinary cooperation”[Title/Abstract] OR “multi disciplinary cooperation”[Title/Abstract] OR “multidisciplinary patient care”[Title/Abstract] OR “team based care”[Title/Abstract] OR “collaborative care”[Title/Abstract] OR “comanaged care”[Title/Abstract] OR “co managed care”[Title/Abstract] OR “shared care”[Title/Abstract] OR “shared model”[Title/Abstract] OR “shared models”[Title/Abstract] OR “interdisciplinary patient care”[Title/Abstract] OR “interdisciplinary care model*”[Title/Abstract] OR “interorganizational coordination”[Title/Abstract] OR “inter organizational coordination”[Title/Abstract] OR “Family Management Framework”[Title/Abstract] OR “home management”[Title/Abstract]) |
| #2 | Multiple chronic conditions | ("chronic condition*"[Title/Abstract] OR “co morbidit*”[Title/Abstract] OR comorbidit*[Title/Abstract] OR “multi-morbidit*”[Title/Abstract] OR multimorbidity*[Title/Abstract] OR "chronic illness*"[Title/Abstract] OR "chronic disease*"[Title/Abstract] OR polymorbidity[Title/Abstract] OR polymorbidities[Title/Abstract] OR “poly morbidity”[Title/Abstract] OR “poly morbidities”[Title/Abstract] OR diabetes[Title/Abstract] OR diabetic*[Title/Abstract] OR prediabet*[Title/Abstract] OR "cardiovascular disease*"[Title/Abstract] OR “cardiometabolic disease*”[Title/Abstract] OR “cardiometabolic condition*”[Title/Abstract] OR “cardiovascular condition*”[Title/Abstract] OR hypertension[Title/Abstract] OR "high blood pressure"[Title/Abstract] OR “coronary artery disease*”[Title/Abstract] OR “heart failure”[Title/Abstract] OR “peripheral vascular disease*”[Title/Abstract] OR “heart disease*”[Title/Abstract] OR “vascular disease*”[Title/Abstract] OR hyperlipidemia[Title/Abstract] OR hypercholesterolemia[Title/Abstract] OR dyslipidemia[Title/Abstract] OR "high cholesterol"[Title/Abstract] OR stroke[Title/Abstract] OR "cerebrovascular accident*"[Title/Abstract] OR “chronic respiratory disease*”[Title/Abstract] OR “chronic obstructive pulmonary disease*”[Title/Abstract] OR COPD[Title/Abstract] OR “chronic obstructive airway disease”[Title/Abstract] OR asthma[Title/Abstract] OR “cognitive decline”[Title/Abstract] OR Alzheimer*[Title/Abstract] OR dementia*[Title/Abstract] OR "chronic kidney disease*"[Title/Abstract] OR "chronic kidney failure"[Title/Abstract] OR “chronic liver disease*”[Title/Abstract] OR “chronic liver failure”[Title/Abstract] OR cirrhosis[Title/Abstract] OR "chronic renal disease*"[Title/Abstract] OR "chronic renal insufficienc*"[Title/Abstract] OR “end stage renal disease*”[Title/Abstract] OR osteoarthritis[Title/Abstract] OR “chronic musculoskeletal disease*”[Title/Abstract] OR “systemic lupus erythematosus”[Title/Abstract] OR "autoimmune disease*"[Title/Abstract] OR "auto immune disease*"[Title/Abstract] OR obesity[Title/Abstract] OR obese[Title/Abstract] OR Obesity[Majr:Noexp] OR cancer[Title/Abstract] OR cancers[Title/Abstract] OR "Chronic Disease"[Majr:Noexp] OR "Multiple Chronic Conditions"[Majr] OR "Stroke"[Majr:Noexp] OR "Diabetes Mellitus, Type 1"[Majr:Noexp] OR "Diabetes Mellitus, Type 2"[Majr:Noexp] OR "Kidney Failure, Chronic"[Majr:Noexp] OR "Renal Insufficiency, Chronic"[Majr:Noexp] OR "Hypertension"[Majr:Noexp] OR "Hyperlipidemias"[Majr:Noexp] OR "Cardiovascular Diseases"[Majr:Noexp] OR "Heart Diseases"[Majr:Noexp] OR "Dyslipidemias"[Majr:Noexp] OR Asthma[Majr:Noexp] OR "Alzheimer Disease"[Majr:Noexp] OR "Dementia"[Majr:Noexp] OR "Osteoarthritis"[Majr:Noexp] OR "Lupus Erythematosus, Systemic"[Majr:Noexp] OR Liver Cirrhosis[Majr:Noexp] OR "Pulmonary Disease, Chronic Obstructive"[Majr:Noexp] OR "Autoimmune Diseases"[Majr:Noexp] OR depression[Title/Abstract] OR “Depressive disorder*”[Title/Abstract] OR “bipolar disorder”[Title/Abstract] OR schizophrenia[Title/Abstract] OR schizophrenic[Title/Abstract] OR “mental disorder*”[Title/Abstract] OR “mental health disorder”[Title/Abstract] OR “mental health disorders”[Title/Abstract] OR "Bipolar Disorder"[Majr] OR "Schizophrenia"[Majr:Noexp] OR "Depression"[Majr] OR "Depressive Disorder"[Majr:Noexp] OR “Mental Disorders”[Majr:Noexp]) |
| #3 | United States | (((Northwest*[Title/Abstract] OR northeast*[Title/Abstract] OR southwest*[Title/Abstract] OR southeast*[Title/Abstract] OR midwest*[Title/Abstract] OR southern[Title/Abstract] OR northern[Title/Abstract] OR "new england"[Title/Abstract] OR "mid atlantic"[Title/Abstract] OR "Great lakes region"[Title/Abstract] OR "appalachian region"[Title/Abstract] OR “pacific northwest”[Title/Abstract]) AND (USA[Title/Abstract] OR "United States"[Title/Abstract] OR US[Title] OR u.s.[Title] OR u.s.a.[Title/Abstract])) OR ((ALABAMA[Title/Abstract] OR ALASKA[Title/Abstract] OR ARIZONA[Title/Abstract] OR ARKANSAS[Title/Abstract] OR CALIFORNIA[Title/Abstract] OR COLORADO[Title/Abstract] OR CONNECTICUT[Title/Abstract] OR DELAWARE[Title/Abstract] OR FLORIDA[Title/Abstract] OR GEORGIA[Title/Abstract] OR HAWAII[Title/Abstract] OR IDAHO[Title/Abstract] OR ILLINOIS[Title/Abstract] OR INDIANA[Title/Abstract] OR IOWA[Title/Abstract] OR KANSAS[Title/Abstract] OR KENTUCKY[Title/Abstract] OR LOUISIANA[Title/Abstract] OR MAINE[Title/Abstract] OR MARYLAND[Title/Abstract] OR MASSACHUSETTS[Title/Abstract] OR MICHIGAN[Title/Abstract] OR MINNESOTA[Title/Abstract] OR MISSISSIPPI[Title/Abstract] OR MISSOURI[Title/Abstract] OR MONTANA[Title/Abstract] OR NEBRASKA[Title/Abstract] OR NEVADA[Title/Abstract] OR "NEW HAMPSHIRE"[Title/Abstract] OR "NEW JERSEY"[Title/Abstract] OR "NEW MEXICO"[Title/Abstract] OR "NEW YORK"[Title/Abstract] OR "NORTH CAROLINA"[Title/Abstract] OR "NORTH DAKOTA"[Title/Abstract] OR OHIO[Title/Abstract] OR OKLAHOMA[Title/Abstract] OR OREGON[Title/Abstract] OR PENNSYLVANIA[Title/Abstract] OR "RHODE ISLAND"[Title/Abstract] OR "SOUTH CAROLINA"[Title/Abstract] OR "SOUTH DAKOTA"[Title/Abstract] OR TENNESSEE[Title/Abstract] OR TEXAS[Title/Abstract] OR UTAH[Title/Abstract] OR VERMONT[Title/Abstract] OR VIRGINIA[Title/Abstract] OR WASHINGTON[Title/Abstract] OR "WEST VIRGINIA"[Title/Abstract] OR WISCONSIN[Title/Abstract] OR WYOMING[Title/Abstract] OR USA[Title/Abstract] OR "United States"[Title/Abstract] OR US[Title] OR u.s.[Title] OR u.s.a.[Title/Abstract] OR American*[Title/Abstract] OR GUAM[Title/Abstract] OR "PUERTO RICO"[Title/Abstract] OR "VIRGIN ISLANDS"[Title/Abstract] OR "American Samoa"[Title/Abstract] OR "Northern Mariana Islands"[Title/Abstract] OR "New York City"[Title/Abstract] OR "Los Angeles"[Title/Abstract] OR Houston[Title/Abstract] OR Dallas[Title/Abstract] OR Miami[Title/Abstract] OR Chicago[Title/Abstract] OR Dallas[Title/Abstract] OR Detroit[Title/Abstract] OR Seattle[Title/Abstract] OR "San Francisco"[Title/Abstract] OR Sacramento[Title/Abstract] OR "San Diego"[Title/Abstract] OR Boston[Title/Abstract] OR "Washington DC"[Title/Abstract] OR “district of columbia”[Title/Abstract] OR Baltimore[Title/Abstract] OR Pittsburgh[Title/Abstract] OR Philadelphia[Title/Abstract] OR Denver[Title/Abstract] OR Phoenix[Title/Abstract] OR "New Orleans"[Title/Abstract] OR ALABAMA[Mesh] OR ALASKA[Mesh] OR ARIZONA[Mesh] OR ARKANSAS[Mesh] OR CALIFORNIA[Mesh] OR COLORADO[Mesh] OR CONNECTICUT[Mesh] OR DELAWARE[Mesh] OR FLORIDA[Mesh] OR GEORGIA[Mesh] OR HAWAII[Mesh] OR IDAHO[Mesh] OR ILLINOIS[Mesh] OR INDIANA[Mesh] OR IOWA[Mesh] OR KANSAS[Mesh] OR KENTUCKY[Mesh] OR LOUISIANA[Mesh] OR MAINE[Mesh] OR MARYLAND[Mesh] OR MASSACHUSETTS[Mesh] OR MICHIGAN[Mesh] OR MINNESOTA[Mesh] OR MISSISSIPPI[Mesh] OR MISSOURI[Mesh] OR MONTANA[Mesh] OR NEBRASKA[Mesh] OR NEVADA[Mesh] OR "NEW HAMPSHIRE"[Mesh] OR "NEW JERSEY"[Mesh] OR "NEW MEXICO"[Mesh] OR "NEW YORK"[Mesh] OR "NORTH CAROLINA"[Mesh] OR "NORTH DAKOTA"[Mesh] OR OHIO[Mesh] OR OKLAHOMA[Mesh] OR OREGON[Mesh] OR PENNSYLVANIA[Mesh] OR "RHODE ISLAND"[Mesh] OR "SOUTH CAROLINA"[Mesh] OR "SOUTH DAKOTA"[Mesh] OR TENNESSEE[Mesh] OR TEXAS[Mesh] OR UTAH[Mesh] OR VERMONT[Mesh] OR VIRGINIA[Mesh] OR WASHINGTON[Mesh] OR "WEST VIRGINIA"[Mesh] OR WISCONSIN[Mesh] OR WYOMING[Mesh] OR "United States"[Mesh] OR "American Samoa"[Mesh] OR GUAM[Mesh] OR "PUERTO RICO"[Mesh] OR "United States VIRGIN ISLANDS"[Mesh] OR "New York City"[Mesh] OR "District of Columbia"[Mesh] OR "Philadelphia"[Mesh] OR "Baltimore"[Mesh] OR Boston[Mesh] OR Chicago[Mesh] OR "Los Angeles"[Mesh] OR "New Orleans"[Mesh] OR "San Francisco"[Mesh] OR Appalachian Region[Mesh] OR Great Lakes Region[Mesh] OR Mid-Atlantic Region[Mesh] OR Midwestern United States[Mesh] OR New England[Mesh] OR Northwestern United States[Mesh] OR Pacific States[Mesh] OR Southeastern United States[Mesh] OR Southwestern United States[Mesh]) NOT dollar*[Title/Abstract])) |
| #4 |  | #1 AND #2 AND #3 |
| #5 | Limits Applied: Publication year, Language | #4 AND ("2016/01/01"[PDAT] : "2021/12/31"[PDAT]) AND English[lang] |
| #6 | Limit Applied: Exclude Publication type | #5 NOT (letter[Publication Type] OR editorial[Publication Type] OR comment[Publication Type] OR news[Publication Type] OR "Congress"[Publication Type] OR "Consensus Development Conference"[Publication Type] OR editorial[Title/Abstract] OR commentary[Title/Abstract] OR “conference abstract*”[Title/Abstract] OR “conference proceeding*”[Title/Abstract] OR symposium*[Title/Abstract] OR "Published Erratum"[Publication Type] OR errata[Title/Abstract] OR erratum[Title/Abstract] OR corrigenda[Title/Abstract] OR corrigendum[Title/Abstract] OR protocol[Title] OR protocols[Title] OR “meta-analysis”[Title/Abstract] OR “meta-analyses”[Title/Abstract] OR metanalyses[Title/Abstract] OR metanalysis[Title/Abstract] OR “meta analyses”[Title/Abstract] OR “meta analysis”[Title/Abstract] OR "Review"[Publication Type] OR “systematic review*”[Title/Abstract] OR "Systematic Review"[Publication Type] OR "Meta-Analysis" [Publication Type] OR "Network Meta-Analysis"[Mesh] OR "Review"[Publication Type]) |

**Notes:** The limits for language (English) and publication year (2016–2021) were applied to the main search using the filters available in PubMed. The keywords were searched in the title and abstract fields in PubMed (i.e., [Title/Abstract]), text field (i.e., [Text Word]), title (i.e., [Title]), and the controlled vocabulary terms are indicated with [Mesh]; the Mesh terms were also searched as Major Mesh and explosion turned off (i.e., [Majr:Noexp]). Terms searched in the [Text Word] field are searched in the title, abstract, keywords, and MeSH fields. Phrases were enclosed in quotation marks to force the searching of the exact terms in order presented. To these results, the search strategy to exclude specific publication types was used. No other limits were applied to the searches.

**Database:** Embase
**Platform:** Elsevier

**Database Date Coverage:** 1947–present
**Date Searched:** March 28, 2022
**Date Limits:** 2016–2021
**Other Limits/Filters:** Language: English; Source: Embase; Excludes: letters, editorials, commentaries, conference abstracts, reviews, meta-analyses

| **Set** | **Concept** | **Search Strategy** |
| --- | --- | --- |
| #1 | Healthcare model | ('healthcare model*':ti,ab OR 'health care model*':ti,ab OR 'healthcare delivery model*':ti,ab OR 'health care delivery model*':ti,ab OR 'delivery model*':ti,ab OR 'delivery care model*':ti,ab OR 'care model':ti,ab OR 'care models':ti,ab OR 'care delivery model*':ti,ab OR 'multispecialty clinic*':ti,ab OR 'multi-specialty clinic*':ti,ab OR 'integrated care':ti,ab OR 'integrated delivery system*':ti,ab OR 'integrated delivery of healthcare':ti,ab OR 'integrated delivery of health care':ti,ab OR 'integrated practice':ti,ab OR 'community health plan*':ti,ab OR 'value based care':ti,ab OR 'value based healthcare':ti,ab OR 'value based health care':ti,ab OR 'value based payment*':ti,ab OR 'Nurse Care Model*':ti,ab OR 'nurse led':ti,ab OR 'nurse management model*':ti,ab OR 'Chronic Illness Self-management':ti,ab OR 'chronic condition self management':ti,ab OR 'chronic disease self management':ti,ab OR 'chronic care model*':ti,ab OR 'chronic care initiative*':ti,ab OR 'community based transition*':ti,ab OR 'home based model*':ti,ab OR 'home based care':ti,ab OR 'patient centered care':ti,ab OR 'patient centered model*':ti,ab OR 'patient centered medical home*':ti,ab OR 'accountable care organization*':ti,ab OR 'managed care model*':ti,ab OR 'sub specialty care':ti,ab OR 'subspecialty care':ti,ab OR telemedicine:ti,ab OR telehealth:ti,ab OR 'care coordination':ti,ab OR 'coordinated care':ti,ab OR 'multidisciplinary care':ti,ab OR 'multi disciplinary care':ti,ab OR 'multidisciplinary specialist care':ti,ab OR 'multidisciplinary cooperation':ti,ab OR 'multi disciplinary cooperation':ti,ab OR 'multidisciplinary patient care':ti,ab OR 'team based care':ti,ab OR 'collaborative care':ti,ab OR 'comanaged care':ti,ab OR 'co managed care':ti,ab OR 'shared care':ti,ab OR 'shared model':ti,ab OR 'shared models':ti,ab OR 'interdisciplinary patient care':ti,ab OR 'interdisciplinary care model*':ti,ab OR 'interorganizational coordination':ti,ab OR 'inter organizational coordination':ti,ab OR 'Family Management Framework':ti,ab OR 'home management':ti,ab OR 'patient centered medical home'/mj OR 'health care planning'/mj OR 'value based care'/exp/mj OR 'accountable care organization'/mj OR 'integrated care'/mj OR 'chronic care model'/mj OR 'telemedicine'/mj OR 'health care'/mj OR 'patient care planning'/mj OR 'self management support'/mj OR 'shared care'/mj OR 'multidisciplinary care'/mj OR 'coordinated care'/mj) |
| #2 | Multiple chronic conditions | ('chronic condition*':ti,ab OR 'co morbidit*':ti,ab OR comorbidit*:ti,ab OR 'multi-morbidit*':ti,ab OR multimorbidity*:ti,ab OR 'chronic illness*':ti,ab OR 'chronic disease*':ti,ab OR polymorbidity:ti,ab OR polymorbidities:ti,ab OR 'poly morbidity':ti,ab OR 'poly morbidities':ti,ab OR diabetes:ti,ab OR diabetic*:ti,ab OR prediabet*:ti,ab OR 'cardiovascular disease*':ti,ab OR 'cardiometabolic disease*':ti,ab OR 'cardiometabolic condition*':ti,ab OR 'cardiovascular condition*':ti,ab OR hypertension:ti,ab OR 'high blood pressure':ti,ab OR 'coronary artery disease*':ti,ab OR 'heart failure':ti,ab OR 'peripheral vascular disease*':ti,ab OR 'heart disease*':ti,ab OR 'vascular disease*':ti,ab OR hyperlipidemia:ti,ab OR hypercholesterolemia:ti,ab OR dyslipidemia:ti,ab OR 'high cholesterol':ti,ab OR stroke:ti,ab OR 'cerebrovascular accident*':ti,ab OR 'chronic respiratory disease*':ti,ab OR 'chronic obstructive pulmonary disease*':ti,ab OR COPD:ti,ab OR 'chronic obstructive airway disease':ti,ab OR asthma:ti,ab OR 'cognitive decline':ti,ab OR Alzheimer*:ti,ab OR dementia*:ti,ab OR 'chronic kidney disease*':ti,ab OR 'chronic kidney failure':ti,ab OR 'chronic liver disease*':ti,ab OR 'chronic liver failure':ti,ab OR cirrhosis:ti,ab OR 'chronic renal disease*':ti,ab OR 'chronic renal insufficienc*':ti,ab OR 'end stage renal disease*':ti,ab OR osteoarthritis:ti,ab OR 'chronic musculoskeletal disease*':ti,ab OR 'systemic lupus erythematosus':ti,ab OR 'autoimmune disease*':ti,ab OR 'auto immune disease*':ti,ab OR obesity:ti,ab OR obese:ti,ab OR cancer:ti,ab OR cancers:ti,ab OR depression:ti,ab OR 'depressive disorder*':ti,ab OR 'bipolar disorder':ti,ab OR schizophrenia:ti,ab OR schizophrenic:ti,ab OR 'mental disorder*':ti,ab OR 'mental health disorder':ti,ab OR 'mental health disorders':ti,ab OR 'insulin dependent diabetes mellitus'/mj OR 'non insulin dependent diabetes mellitus'/mj OR 'diabetes mellitus'/mj OR 'obesity'/mj OR 'cerebrovascular accident'/mj OR 'hypertension'/mj OR 'hyperlipidemia'/mj OR 'dyslipidemia'/mj OR 'chronic kidney failure'/mj OR 'cardiovascular disease'/mj OR 'heart disease'/mj OR 'asthma'/mj OR 'dementia'/mj OR 'Alzheimer disease'/mj OR 'osteoarthritis'/mj OR 'liver cirrhosis'/mj OR 'chronic obstructive lung disease'/mj OR 'autoimmune disease'/mj OR 'systemic lupus erythematosus'/mj OR 'bipolar disorder'/mj OR 'schizophrenia'/mj OR 'mental disease'/mj OR 'depression'/mj OR 'multiple chronic conditions'/exp OR 'comorbidity'/mj OR 'chronic disease'/exp/mj) |
| #3 | United States | (((Northwest*:ti,ab OR northeast*:ti,ab OR southwest*:ti,ab OR southeast*:ti,ab OR midwest*:ti,ab OR southern:ti,ab OR northern:ti,ab OR 'new england':ti,ab OR 'mid atlantic':ti,ab OR 'Great lakes region':ti,ab OR 'pacific northwest':ti,ab OR 'appalachian region':ti,ab) AND (USA:ti,ab OR 'United States':ti,ab OR US:ti OR u.s.:ti OR u.s.a.:ti,ab)) OR ((ALABAMA:ti,ab OR ALASKA:ti,ab OR ARIZONA:ti,ab OR ARKANSAS:ti,ab OR CALIFORNIA:ti,ab OR COLORADO:ti,ab OR CONNECTICUT:ti,ab OR DELAWARE:ti,ab OR FLORIDA:ti,ab OR GEORGIA:ti,ab OR HAWAII:ti,ab OR IDAHO:ti,ab OR ILLINOIS:ti,ab OR INDIANA:ti,ab OR IOWA:ti,ab OR KANSAS:ti,ab OR KENTUCKY:ti,ab OR LOUISIANA:ti,ab OR MAINE:ti,ab OR MARYLAND:ti,ab OR MASSACHUSETTS:ti,ab OR MICHIGAN:ti,ab OR MINNESOTA:ti,ab OR MISSISSIPPI:ti,ab OR MISSOURI:ti,ab OR MONTANA:ti,ab OR NEBRASKA:ti,ab OR NEVADA:ti,ab OR 'NEW HAMPSHIRE':ti,ab OR 'NEW JERSEY':ti,ab OR 'NEW MEXICO':ti,ab OR 'NEW YORK':ti,ab OR 'NORTH CAROLINA':ti,ab OR 'NORTH DAKOTA':ti,ab OR OHIO:ti,ab OR OKLAHOMA:ti,ab OR OREGON:ti,ab OR PENNSYLVANIA:ti,ab OR 'RHODE ISLAND':ti,ab OR 'SOUTH CAROLINA':ti,ab OR 'SOUTH DAKOTA':ti,ab OR TENNESSEE:ti,ab OR TEXAS:ti,ab OR UTAH:ti,ab OR VERMONT:ti,ab OR VIRGINIA:ti,ab OR WASHINGTON:ti,ab OR 'WEST VIRGINIA':ti,ab OR WISCONSIN:ti,ab OR WYOMING:ti,ab OR USA:ti,ab OR 'United States':ti,ab OR US:ti OR u.s.:ti OR u.s.a.:ti,ab OR American*:ti,ab OR GUAM:ti,ab OR 'PUERTO RICO':ti,ab OR 'VIRGIN ISLANDS':ti,ab OR 'American Samoa':ti,ab OR 'Northern Mariana Islands':ti,ab OR 'New York City':ti,ab OR 'Los Angeles':ti,ab OR Houston:ti,ab OR Dallas:ti,ab OR Miami:ti,ab OR Chicago:ti,ab OR Dallas:ti,ab OR Detroit:ti,ab OR Seattle:ti,ab OR 'San Francisco':ti,ab OR Sacramento:ti,ab OR 'San Diego':ti,ab OR Boston:ti,ab OR 'Washington DC':ti,ab OR Baltimore:ti,ab OR Pittsburgh:ti,ab OR Philadelphia:ti,ab OR Denver:ti,ab OR Phoenix:ti,ab OR 'New Orleans':ti,ab OR ALABAMA/exp OR ALASKA/exp OR ARIZONA/exp OR ARKANSAS/exp OR CALIFORNIA/exp OR COLORADO/exp OR CONNECTICUT/exp OR DELAWARE/exp OR FLORIDA/exp OR GEORGIA/exp OR HAWAII/exp OR IDAHO/exp OR ILLINOIS/exp OR INDIANA/exp OR IOWA/exp OR KANSAS/exp OR KENTUCKY/exp OR LOUISIANA/exp OR MAINE/exp OR MARYLAND/exp OR MASSACHUSETTS/exp OR MICHIGAN/exp OR MINNESOTA/exp OR MISSISSIPPI/exp OR MISSOURI/exp OR MONTANA/exp OR NEBRASKA/exp OR NEVADA/exp OR 'NEW HAMPSHIRE'/exp OR 'NEW JERSEY'/exp OR 'NEW MEXICO'/exp OR 'NEW YORK'/exp OR 'NORTH CAROLINA'/exp OR 'NORTH DAKOTA'/exp OR OHIO/exp OR OKLAHOMA/exp OR OREGON/exp OR PENNSYLVANIA/exp OR 'RHODE ISLAND'/exp OR 'SOUTH CAROLINA'/exp OR 'SOUTH DAKOTA'/exp OR TENNESSEE/exp OR TEXAS/exp OR UTAH/exp OR VERMONT/exp OR VIRGINIA/exp OR WASHINGTON/exp OR 'WEST VIRGINIA'/exp OR WISCONSIN/exp OR WYOMING/exp OR 'United States'/exp OR 'American Samoa'/exp OR GUAM/exp OR 'PUERTO RICO'/exp OR 'United states VIRGIN ISLANDS'/exp OR 'New York City'/exp OR 'District of Columbia'/exp OR 'Philadelphia'/exp OR 'Baltimore'/exp OR Boston/exp OR Chicago/exp OR 'Los Angeles'/exp OR 'New Orleans'/exp OR 'San Francisco'/exp OR ‘Appalachian Region’:ti,ab OR ‘Great Lakes Region’:ti,ab OR ‘Mid-Atlantic Region’:ti,ab OR ‘Midwestern United States’:ti,ab OR ‘New England’:ti,ab OR ‘Northwestern United States’:ti,ab OR ‘Pacific States’:ti,ab OR ‘Southeastern United States’:ti,ab OR ‘Southwestern United States’:ti,ab) NOT dollar*:ti,ab)) |
| #4 |  | #1 AND #2 AND #3 |
| #5 | Limits Applied: Publication year, Source, Language | #4 AND ([embase]/lim AND [english]/lim AND [2016-2021]/py) |
| #6 | Limit Applied: Exclude Publication type | #5 NOT ([conference abstract]/lim OR [conference paper]/lim OR [conference review]/lim OR [data papers]/lim OR [editorial]/lim OR [erratum]/lim OR [letter]/lim OR [note]/lim OR [short survey]/lim OR 'conference abstract'/exp OR 'conference paper'/exp OR 'data paper'/exp OR 'editorial'/exp OR 'letter'/exp OR 'erratum'/exp OR 'note'/exp OR 'short survey'/exp OR corrigenda:ti,ab OR corrigendum:ti,ab OR erratum:ti,ab OR errata:ti,ab OR protocol*:ti,ab OR ‘conference abstract*’:ti,ab OR ‘conference proceeding*’:ti,ab OR symposium*:ti,ab OR letter:ti,ab OR editorial:ti,ab OR commentary:ti,ab OR review:ti,ab OR [systematic review]/lim OR 'review'/exp OR 'systematic review'/exp OR [review]/lim OR 'meta analysis':ti,ab OR 'meta analyses':ti,ab OR 'meta-analysis':ti,ab OR 'meta-analyses':ti,ab OR metanalysis:ti,ab OR metanalyses:ti,ab) |

**Notes:** The limits for language (English) and publication year (2016–2021) were applied to the main search using the filters available in Embase. The keywords were searched in the title and abstract fields (i.e., :ti,ab), title (i.e., :ti), and the controlled vocabulary terms are indicated with /mj or /exp; the EMTREE terms were also searched as Major (i.e., /mj) and explosion turned off (i.e., /exp). Phrases were enclosed in quotation marks to force the searching of the exact terms in order presented. To these results, the search strategy to exclude specific publication types was used. No other limits were applied to the searches.

**Database:** Scopus
**Platform:** Elsevier
**Database Date Coverage:** 1788–present

**Date Searched:** March 28, 2022
**Date Limits:** 2016–2021
**Other Limits/Filters:** Language: English; Excludes: letters, editorials, commentaries, conference abstracts, reviews, meta-analyses

| **Set** | **Concept** | **Search Strategy** |
| --- | --- | --- |
| #1 | Healthcare model | Title-Abs-Key(({healthcare model*} OR {health care model*} OR {healthcare delivery model*} OR {health care delivery model*} OR {delivery model*} OR {delivery care model*} OR {care model} OR {care models} OR {care delivery model*} OR {multispecialty clinic*} OR {multi-specialty clinic*} OR {integrated care} OR {integrated delivery system*} OR {integrated delivery of healthcare} OR {integrated delivery of health care} OR {integrated practice} OR {community health plan*} OR {value based care} OR {value based healthcare} OR {value based health care} OR {value based payment*} OR {Nurse Care Model*} OR {nurse led} OR {nurse management model*} OR {Chronic Illness Self-management} OR {chronic condition self management} OR {chronic disease self management} OR {chronic care model*} OR {chronic care initiative*} OR {community based transition*} OR {home based model*} OR {home based care} OR {patient centered care} OR {patient centered model*} OR {patient centered medical home*} OR {accountable care organization*} OR {managed care model*} OR {sub specialty care} OR {subspecialty care} OR telemedicine OR telehealth OR {care coordination} OR {coordinated care} OR {multidisciplinary care} OR {multi disciplinary care} OR {multidisciplinary specialist care} OR {multidisciplinary cooperation} OR {multi disciplinary cooperation} OR {multidisciplinary patient care} OR {team based care} OR {collaborative care} OR {comanaged care} OR {co managed care} OR {shared care} OR {shared model} OR {shared models} OR {interdisciplinary patient care} OR {interdisciplinary care model*} OR {interorganizational coordination} OR {inter organizational coordination} OR {Family Management Framework} OR {home management})) |
| #2 | Multiple chronic conditions | Title-Abs-Key(({chronic condition*} OR {co morbidit*} OR comorbidit* OR {multi-morbidit*} OR multimorbidity* OR {chronic illness*} OR {chronic disease*} OR polymorbidity OR polymorbidities OR {poly morbidity} OR {poly morbidities} OR diabetes OR diabetic* OR prediabet* OR {cardiovascular disease*} OR {cardiometabolic disease*} OR {cardiometabolic condition*} OR {cardiovascular condition*} OR hypertension OR {high blood pressure} OR {coronary artery disease*} OR {heart failure} OR {peripheral vascular disease*} OR {heart disease*} OR {vascular disease*} OR hyperlipidemia OR hypercholesterolemia OR dyslipidemia OR {high cholesterol} OR stroke OR {cerebrovascular accident*} OR {chronic respiratory disease*} OR {chronic obstructive pulmonary disease*} OR COPD OR {chronic obstructive airway disease} OR asthma OR {cognitive decline} OR Alzheimer* OR dementia* OR {chronic kidney disease*} OR {chronic kidney failure} OR {chronic liver disease*} OR {chronic liver failure} OR cirrhosis OR {chronic renal disease*} OR {chronic renal insufficienc*} OR {end stage renal disease*} OR osteoarthritis OR {chronic musculoskeletal disease*} OR {systemic lupus erythematosus} OR {autoimmune disease*} OR {auto immune disease*} OR obesity OR obese OR cancer OR cancers OR depression OR {Depressive disorder*} OR {bipolar disorder} OR schizophrenia OR schizophrenic OR {mental disorder*} OR {mental health disorder} OR {mental health disorders})) |
| #3 | United States | Title-Abs-Key((((Northwest* OR northeast* OR southwest* OR southeast* OR midwest* OR southern OR northern OR {new england} OR {mid atlantic} OR {Great lakes region} OR {appalachian region}) AND (USA OR {United States} OR US OR u.s. OR u.s.a.)) OR ((ALABAMA OR ALASKA OR ARIZONA OR ARKANSAS OR CALIFORNIA OR COLORADO OR CONNECTICUT OR DELAWARE OR FLORIDA OR GEORGIA OR HAWAII OR IDAHO OR ILLINOIS OR INDIANA OR IOWA OR KANSAS OR KENTUCKY OR LOUISIANA OR MAINE OR MARYLAND OR MASSACHUSETTS OR MICHIGAN OR MINNESOTA OR MISSISSIPPI OR MISSOURI OR MONTANA OR NEBRASKA OR NEVADA OR {NEW HAMPSHIRE} OR {NEW JERSEY} OR {NEW MEXICO} OR {NEW YORK} OR {NORTH CAROLINA} OR {NORTH DAKOTA} OR OHIO OR OKLAHOMA OR OREGON OR PENNSYLVANIA OR {RHODE ISLAND} OR {SOUTH CAROLINA} OR {SOUTH DAKOTA} OR TENNESSEE OR TEXAS OR UTAH OR VERMONT OR VIRGINIA OR WASHINGTON OR {WEST VIRGINIA} OR WISCONSIN OR WYOMING OR USA OR {United States} OR US OR u.s. OR u.s.a. OR American* OR GUAM OR {PUERTO RICO} OR {VIRGIN ISLANDS} OR {American Samoa} OR {Northern Mariana Islands} OR {New York City} OR {Los Angeles} OR Houston OR Dallas OR Miami OR Chicago OR Dallas OR Detroit OR Seattle OR {San Francisco} OR Sacramento OR {San Diego} OR Boston OR {Washington DC} OR Baltimore OR Pittsburgh OR Philadelphia OR Denver OR Phoenix OR {New Orleans} OR {Appalachian Region} OR {Great Lakes Region} OR {Mid-Atlantic Region} OR {Midwestern United States} OR {New England} OR {Northwestern United States} OR {Pacific States} OR {Southeastern United States} OR {Southwestern United States}) AND NOT dollar*) )) |
| #4 |  | #1 AND #2 AND #3 |
| #5 | Limits Applied: Publication year, Language | #4 AND ( LIMIT-TO ( PUBYEAR,2021) OR LIMIT-TO ( PUBYEAR,2020) OR LIMIT-TO ( PUBYEAR,2019) OR LIMIT-TO ( PUBYEAR,2018) OR LIMIT-TO ( PUBYEAR,2017) OR LIMIT-TO ( PUBYEAR,2016) ) AND ( LIMIT-TO ( LANGUAGE,"English" ) ) AND ( EXCLUDE ( LANGUAGE , "Spanish" ) OR EXCLUDE ( LANGUAGE , "French" ) OR EXCLUDE ( LANGUAGE , "German" ) OR EXCLUDE ( LANGUAGE , "Greek" ) OR EXCLUDE ( LANGUAGE , "Italian" ) OR EXCLUDE ( LANGUAGE , "Portuguese" ) ) |
| #6 | Limit Applied: Exclude Publication type | #5 NOT ( EXCLUDE ( DOCTYPE , "re" ) OR EXCLUDE ( DOCTYPE , "no" ) OR EXCLUDE ( DOCTYPE , "ch" ) OR EXCLUDE ( DOCTYPE , "ed" ) OR EXCLUDE ( DOCTYPE , "er" ) OR EXCLUDE ( DOCTYPE , "le" ) ) AND ( LIMIT-TO ( DOCTYPE , "ar" ) ) |

**Notes:** The limits for language (English) and publication year (2016–2021) were applied to the main search using the filters available in Scopus. The keywords were searched in the title, abstract and keywords fields (i.e., Title-Abs-Key). Phrases were enclosed in quotation marks to force the searching of the exact terms in order presented. To these results, the search strategy to exclude specific publication types was used. No other limits were applied to the searches.

**Database:** CINAHL Plus
**Platform:** EBSCOhost

**Database Date Coverage:** 1937–present
**Date Searched:** March 28, 2022
**Date Limits:** 2016–2021
**Other Limits/Filters:** English; Excludes: letters, editorials, commentaries, conference abstracts, reviews, meta-analyses

| **Set** | **Concept** | **Search Strategy** |
| --- | --- | --- |
| #1 | Healthcare model | Title: ("healthcare model*" OR "health care model*" OR “healthcare delivery model*” OR “health care delivery model*” OR “delivery model*” OR “delivery care model*” OR “care model” OR “care models” OR “care delivery model*” OR “multispecialty clinic*” OR “multi-specialty clinic*” OR "integrated care" OR "integrated delivery system*" OR “integrated delivery of healthcare” OR “integrated delivery of health care” OR “integrated practice” OR "community health plan*" OR "value based care" OR "value based healthcare" OR "value based health care" OR “value based payment*” OR “Nurse Care Model*” OR “nurse led” OR "nurse management model*" OR “Chronic Illness Self-management” OR “chronic condition self management” OR “chronic disease self management” OR "chronic care model*" OR “chronic care initiative*” OR "community based transition*" OR "home based model*" OR “home based care” OR "patient centered care" OR “patient centered model*” OR "patient centered medical home*" OR "accountable care organization*" OR "managed care model*" OR “sub specialty care” OR “subspecialty care” OR telemedicine OR telehealth OR “care coordination” OR “coordinated care” OR “multidisciplinary care” OR “multi disciplinary care” OR “multidisciplinary specialist care” OR “multidisciplinary cooperation” OR “multi disciplinary cooperation” OR “multidisciplinary patient care” OR “team based care” OR “collaborative care” OR “comanaged care” OR “co managed care” OR “shared care” OR “shared model” OR “shared models” OR “interdisciplinary patient care” OR “interdisciplinary care model*” OR “interorganizational coordination” OR “inter organizational coordination” OR “Family Management Framework” OR “home management”) |
| #2 | Healthcare model | Abstract: ("healthcare model*" OR "health care model*" OR “healthcare delivery model*” OR “health care delivery model*” OR “delivery model*” OR “delivery care model*” OR “care model” OR “care models” OR “care delivery model*” OR “multispecialty clinic*” OR “multi-specialty clinic*” OR "integrated care" OR "integrated delivery system*" OR “integrated delivery of healthcare” OR “integrated delivery of health care” OR “integrated practice” OR "community health plan*" OR "value based care" OR "value based healthcare" OR "value based health care" OR “value based payment*” OR “Nurse Care Model*” OR “nurse led” OR "nurse management model*" OR “Chronic Illness Self-management” OR “chronic condition self management” OR “chronic disease self management” OR "chronic care model*" OR “chronic care initiative*” OR "community based transition*" OR "home based model*" OR “home based care” OR "patient centered care" OR “patient centered model*” OR "patient centered medical home*" OR "accountable care organization*" OR "managed care model*" OR “sub specialty care” OR “subspecialty care” OR telemedicine OR telehealth OR “care coordination” OR “coordinated care” OR “multidisciplinary care” OR “multi disciplinary care” OR “multidisciplinary specialist care” OR “multidisciplinary cooperation” OR “multi disciplinary cooperation” OR “multidisciplinary patient care” OR “team based care” OR “collaborative care” OR “comanaged care” OR “co managed care” OR “shared care” OR “shared model” OR “shared models” OR “interdisciplinary patient care” OR “interdisciplinary care model*” OR “interorganizational coordination” OR “inter organizational coordination” OR “Family Management Framework” OR “home management”) |
| #3 | Healthcare model | Exact Major Subject Heading: ((MM "Health Care Delivery, Integrated") OR (MM "Telemedicine") OR (MM "Value-Based Health Care") OR (MM "Patient Centered Care") OR (MM "Accountable Care Organizations") OR (MM "Multidisciplinary Care Team") OR (MM "Self-Management")) |
| #4 | Healthcare model | #1 OR #2 OR #3 |
| #5 | Multiple chronic conditions | Title: ("chronic condition*" OR "co morbidit*" OR comorbidit* OR "multi-morbidit*" OR multimorbidity* OR "chronic illness*" OR "chronic disease*" OR polymorbidity OR polymorbidities OR "poly morbidity" OR "poly morbidities" OR diabetes OR diabetic* OR prediabet* OR "cardiovascular disease*" OR "cardiometabolic disease*" OR "cardiometabolic condition*" OR "cardiovascular condition*" OR hypertension OR "high blood pressure" OR "coronary artery disease*" OR "heart failure" OR "peripheral vascular disease*" OR "heart disease*" OR "vascular disease*" OR hyperlipidemia OR hypercholesterolemia OR dyslipidemia OR "high cholesterol" OR stroke OR "cerebrovascular accident*" OR "chronic respiratory disease*" OR "chronic obstructive pulmonary disease*" OR COPD OR "chronic obstructive airway disease" OR asthma OR "cognitive decline" OR Alzheimer* OR dementia* OR "chronic kidney disease*" OR "chronic kidney failure" OR "chronic liver disease*" OR "chronic liver failure" OR cirrhosis OR "chronic renal disease*" OR "chronic renal insufficienc*" OR "end stage renal disease*" OR osteoarthritis OR "chronic musculoskeletal disease*" OR "systemic lupus erythematosus" OR "autoimmune disease*" OR "auto immune disease*" OR obesity OR obese OR cancer OR cancers OR depression OR "Depressive disorder*" OR "bipolar disorder" OR schizophrenia OR schizophrenic OR "mental disorder*" OR "mental health disorder" OR "mental health disorders") |
| #6 | Multiple chronic conditions | Abstract: ("chronic condition*" OR "co morbidit*" OR comorbidit* OR "multi-morbidit*" OR multimorbidity* OR "chronic illness*" OR "chronic disease*" OR polymorbidity OR polymorbidities OR "poly morbidity" OR "poly morbidities" OR diabetes OR diabetic* OR prediabet* OR "cardiovascular disease*" OR "cardiometabolic disease*" OR "cardiometabolic condition*" OR "cardiovascular condition*" OR hypertension OR "high blood pressure" OR "coronary artery disease*" OR "heart failure" OR "peripheral vascular disease*" OR "heart disease*" OR "vascular disease*" OR hyperlipidemia OR hypercholesterolemia OR dyslipidemia OR "high cholesterol" OR stroke OR "cerebrovascular accident*" OR "chronic respiratory disease*" OR "chronic obstructive pulmonary disease*" OR COPD OR "chronic obstructive airway disease" OR asthma OR "cognitive decline" OR Alzheimer* OR dementia* OR "chronic kidney disease*" OR "chronic kidney failure" OR "chronic liver disease*" OR "chronic liver failure" OR cirrhosis OR "chronic renal disease*" OR "chronic renal insufficienc*" OR "end stage renal disease*" OR osteoarthritis OR "chronic musculoskeletal disease*" OR "systemic lupus erythematosus" OR "autoimmune disease*" OR "auto immune disease*" OR obesity OR obese OR cancer OR cancers OR depression OR "Depressive disorder*" OR "bipolar disorder" OR schizophrenia OR schizophrenic OR "mental disorder*" OR "mental health disorder" OR "mental health disorders") |
| #7 | Multiple chronic conditions | Exact Major Subject Heading: ((MM "Obesity") OR (MM "Chronic Disease") OR (MM "Hypertension") OR (MM "Hyperlipidemia") OR (MM "Hypercholesterolemia") OR (MM "Diabetes Mellitus, Type 1") OR (MM "Diabetes Mellitus, Type 2") OR (MM "Stroke") OR (MM "Kidney Failure, Chronic") OR (MM "Renal Insufficiency, Chronic") OR (MM "Heart Diseases") OR (MM "Cardiovascular Diseases") OR (MM "Schizophrenia") OR (MM "Mental Disorders") OR (MM "Depression") OR (MM "Lupus Erythematosus, Systemic") OR (MM "Autoimmune Diseases") OR (MM "Pulmonary Disease, Chronic Obstructive") OR (MM "Asthma") OR (MM "Liver Cirrhosis") OR (MM "Osteoarthritis") OR (MM "Alzheimer's Disease") OR (MM "Dementia") OR (MM "Comorbidity")) |
| #8 | Multiple chronic conditions | #5 OR #6 OR #7 |
| #9 | United States | Title: (ALABAMA OR ALASKA OR ARIZONA OR ARKANSAS OR CALIFORNIA OR COLORADO OR CONNECTICUT OR DELAWARE OR FLORIDA OR GEORGIA OR HAWAII OR IDAHO OR ILLINOIS OR INDIANA OR IOWA OR KANSAS OR KENTUCKY OR LOUISIANA OR MAINE OR MARYLAND OR MASSACHUSETTS OR MICHIGAN OR MINNESOTA OR MISSISSIPPI OR MISSOURI OR MONTANA OR NEBRASKA OR NEVADA OR "NEW HAMPSHIRE" OR "NEW JERSEY" OR "NEW MEXICO" OR "NEW YORK" OR "NORTH CAROLINA" OR "NORTH DAKOTA" OR OHIO OR OKLAHOMA OR OREGON OR PENNSYLVANIA OR "RHODE ISLAND" OR "SOUTH CAROLINA" OR "SOUTH DAKOTA" OR TENNESSEE OR TEXAS OR UTAH OR VERMONT OR VIRGINIA OR WASHINGTON OR "WEST VIRGINIA" OR WISCONSIN OR WYOMING OR USA OR "United States" OR US OR u.s. OR u.s.a. OR American* OR GUAM OR "PUERTO RICO" OR "VIRGIN ISLANDS" OR "American Samoa" OR "Northern Mariana Islands") |
| #10 | United States | Abstract: (ALABAMA OR ALASKA OR ARIZONA OR ARKANSAS OR CALIFORNIA OR COLORADO OR CONNECTICUT OR DELAWARE OR FLORIDA OR GEORGIA OR HAWAII OR IDAHO OR ILLINOIS OR INDIANA OR IOWA OR KANSAS OR KENTUCKY OR LOUISIANA OR MAINE OR MARYLAND OR MASSACHUSETTS OR MICHIGAN OR MINNESOTA OR MISSISSIPPI OR MISSOURI OR MONTANA OR NEBRASKA OR NEVADA OR "NEW HAMPSHIRE" OR "NEW JERSEY" OR "NEW MEXICO" OR "NEW YORK" OR "NORTH CAROLINA" OR "NORTH DAKOTA" OR OHIO OR OKLAHOMA OR OREGON OR PENNSYLVANIA OR "RHODE ISLAND" OR "SOUTH CAROLINA" OR "SOUTH DAKOTA" OR TENNESSEE OR TEXAS OR UTAH OR VERMONT OR VIRGINIA OR WASHINGTON OR "WEST VIRGINIA" OR WISCONSIN OR WYOMING OR USA OR "United States" OR US OR u.s. OR u.s.a. OR American* OR GUAM OR "PUERTO RICO" OR "VIRGIN ISLANDS" OR "American Samoa" OR "Northern Mariana Islands") |
| #11 | United States | Title: ("New York City" OR "Los Angeles" OR Houston OR Dallas OR Miami OR Chicago OR Dallas OR Detroit OR Seattle OR "San Francisco" OR Sacramento OR "San Diego" OR Boston OR "Washington DC" OR Baltimore OR Pittsburgh OR Philadelphia OR Denver OR Phoenix OR "New Orleans" OR "Appalachian Region" OR "Great Lakes Region" OR "Mid-Atlantic Region" OR "Midwestern United States" OR "New England" OR "Northwestern United States" OR "Pacific States" OR “Pacific Northwest” OR "Southeastern United States" OR "Southwestern United States") |
| #12 | United States | Abstract: ("New York City" OR "Los Angeles" OR Houston OR Dallas OR Miami OR Chicago OR Dallas OR Detroit OR Seattle OR "San Francisco" OR Sacramento OR "San Diego" OR Boston OR "Washington DC" OR Baltimore OR Pittsburgh OR Philadelphia OR Denver OR Phoenix OR "New Orleans" OR "Appalachian Region" OR "Great Lakes Region" OR "Mid-Atlantic Region" OR "Midwestern United States" OR "New England" OR "Northwestern United States" OR "Pacific States" OR “Pacific Northwest” OR "Southeastern United States" OR "Southwestern United States") |
| #13 | United States | Exact Subject Heading: (MH "United States+") OR (MH "United States by Individual State+") OR (MH "United States by Region+") |
| #14 | United States | Title: ((Northwest* OR northeast* OR southwest* OR southeast* OR midwest* OR southern OR northern OR "new england" OR "mid atlantic" OR "Great lakes region" OR "appalachian region" OR “Pacific Northwest”) AND (USA OR "United States" OR US OR u.s. OR u.s.a.)) |
| #15 | United States | Abstract: ((Northwest* OR northeast* OR southwest* OR southeast* OR midwest* OR southern OR northern OR "new england" OR "mid atlantic" OR "Great lakes region" OR "appalachian region" OR “Pacific Northwest”) AND (USA OR "United States" OR US OR u.s. OR u.s.a.)) |
| #16 | United States | #9 OR #10 OR #11 OR #12 OR #13 OR #14 OR #15 OR #16 |
| #17 |  | #4 AND #8 AND #16 |
| #18 | Limits Applied: Publication year, Language, Publication type: | #17 AND Pub Year: 2016-2021 AND Language: English AND Publication Type: Journal Article |
| #19 | Publication type | Exact Subject Heading: ((MH "News" OR (MH "Theses and Dissertations") OR (MH "Protocols") OR (MH "Systematic Review") OR (MH "Scoping Review") OR (MH "Meta Analysis") OR (MH "Congresses and Conferences")) |
| #20 | Publication type | Title: (corrigenda OR corrigendum OR erratum OR errata OR “conference abstract” OR “conference abstracts” OR “conference proceeding” OR “conference proceedings” OR symposium* OR editorial OR commentary OR protocol OR protocols OR “systematic review*” OR “meta analysis” OR “meta analyses” OR metanalysis OR metanalyses) |
| #21 | Publication type | Abstract: (corrigenda OR corrigendum OR erratum OR errata OR “conference abstract” OR “conference abstracts” OR “conference proceeding” OR “conference proceedings” OR symposium* OR editorial OR commentary OR protocol OR protocols OR “systematic review*” OR “meta analysis” OR “meta analyses” OR metanalysis OR metanalyses) |
| #22 |  | #19 OR #20 OR #21 |
| #23 | Limit Applied: Exclude Publication type | #18 NOT #22 |

**Notes:** The limits for language (English) and publication year (2016–2021) were applied to the main search using the filters available in CINAHL Plus. The keywords were searched in the title and abstract fields and the controlled vocabulary terms are indicated Exact Subject Headings (MH) or Exact Major Subject Headings (MM). Phrases were enclosed in quotation marks to force the searching of the exact terms in order presented. To these results, the search strategy to exclude letters, comments, editorials, and retractions was used, and the Publication Type limit “Journal Article” was applied.
